# Interpretable Machine Learning for In-Hospital Mortality Prediction in ICU Patients Using First-24-Hour Routine Vital Signs: A SHAP-Based MIMIC-IV Study

**DOI:** 10.3390/diagnostics16182982

**Published:** 2026-09-15

**Authors:** Abdul Karim, Jinwon Kim, In cheol Jeong

**Affiliations:** 1 Cerebrovascular Disease Research Center, Hallym University, Chuncheon 24252, Republic of Korea; abdullkarim@hallym.ac.kr (A.K.); jinwon.kim@hallym.ac.kr (J.K.); 2 Department of Artificial Intelligence Convergence, Hallym University, Chuncheon 24252, Republic of Korea; 3 Department of Population Health Science and Policy, Icahn School of Medicine at Mount Sinai, New York, NY 10029, USA

**Keywords:** in-hospital mortality, intensive care unit, MIMIC-IV, machine learning, explainable artificial intelligence, SHAP, vital signs, clinical decision support, first-24 h mortality prediction

## Abstract

**Background:** Identification of ICU patients at increased risk of death may support clinical assessment and prioritization of care. However, many mortality prediction approaches incorporate extensive laboratory information, conventional severity scores, or computationally complex models that may limit straightforward interpretation at the bedside. This study investigated whether routinely recorded physiological measurements collected during the first 24 h of ICU care could support an interpretable machine learning approach to subsequent in-hospital mortality prediction. **Methods:** We performed a retrospective analysis of 51,598 adult first ICU admissions from MIMIC-IV using a strict 24 h landmark design. Patients with a missing ICU duration, a documented ICU stay shorter than 24 h, or a documented death occurring on or before the 24 h landmark were excluded. Physiological measurements recorded during the first 24 h were summarized to construct the predictor set. Five classification algorithms—Logistic Regression, Random Forest, XGBoost, LightGBM, and CatBoost—were developed using a stratified 80:20 train–test split. Performance was evaluated using discrimination, precision–recall, calibration, threshold-dependent classification measures, and decision-curve analysis. Bootstrap resampling was used to estimate 95% confidence intervals and pairwise performance differences. Shapley Additive Explanations (SHAPs) were used to interpret the XGBoost model. **Results:** The revised cohort included 46,259 survivors and 5339 patients with an in-hospital mortality outcome. XGBoost achieved the numerically highest AUROC of 0.803 (95% CI: 0.790–0.815) and AUPRC of 0.341 (95% CI: 0.313–0.369), with a Brier score of 0.080 (95% CI: 0.076–0.084). Paired bootstrap comparisons showed that XGBoost had a significantly higher AUROC than Random Forest and LightGBM, but not CatBoost. Its AUPRC was significantly higher than that of Random Forest but did not differ significantly from LightGBM or CatBoost, while its Brier score was significantly lower than that of Random Forest but did not differ significantly from LightGBM or CatBoost. At the default probability threshold of 0.50, XGBoost achieved high specificity (0.990) but low recall (0.091), whereas lowering the threshold increased mortality detection at the cost of additional false-positive classifications. **Conclusions:** Routinely recorded physiological information accumulated during the first 24 h of ICU care supported an interpretable machine learning framework with useful discrimination and probability estimation for subsequent in-hospital mortality. XGBoost achieved the highest numerical performance, although differences among the gradient-boosting models were generally small and not significant for all evaluated metrics. Threshold-sensitivity analysis demonstrated that classification behavior depended strongly on the selected operating threshold. Independent multicenter validation and prospective clinical evaluation are required before routine deployment.

## 1. Introduction

Timely recognition of critically ill patients who are likely to experience adverse outcomes is a persistent challenge in intensive care practice. Patients admitted to intensive care units (ICUs) may undergo substantial physiological changes over relatively short periods, making early recognition of deterioration particularly important for appropriate clinical management. This challenge occurs within healthcare environments that may simultaneously face emergency department (ED) crowding, constrained critical care capacity, and increasingly complex patient populations. Together, these factors reinforce the importance of clinical decision-support approaches capable of providing reliable and timely risk information during the early stages of critical care [1,2,3].

Artificial intelligence (AI) and machine learning (ML) are increasingly being investigated as data-driven approaches for clinical prediction using information contained in electronic health records (EHRs). Rather than depending exclusively on predefined clinical rules, these methods can identify complex relationships among routinely collected patient variables and have been applied to tasks such as mortality estimation, assessment of ICU admission, and patient risk stratification. Growing attention has also been directed toward interpretable ML approaches that combine predictive capability with explanations that can be examined by clinicians and reproduced in subsequent analyses [4,5,6,7]. Despite this progress, practical deployment remains challenging because many existing approaches require broad panels of laboratory measurements, multiple physiological data modalities, or resource-intensive deep learning models that may be difficult to incorporate into routine clinical workflows.

Recent work has explored increasingly sophisticated machine learning strategies for prognostic assessment in intensive care. Hou et al. developed a large-scale framework for identifying ICU patients associated with high levels of healthcare need and expenditure, illustrating how data-driven modeling can support risk stratification within critically ill populations [8]. In a related direction, Long et al. investigated a multi-model fusion approach for 28-day mortality prediction that incorporated subgroup-based validation and clinical risk stratification into the evaluation framework [9]. Although such studies demonstrate the expanding capabilities of machine learning for ICU prognosis, greater modeling complexity does not necessarily translate into easier clinical adoption. Approaches requiring numerous patient variables or elaborate computational pipelines may be difficult to interpret or deploy where data and computational resources are limited. This creates an opportunity for prediction strategies that derive clinically understandable risk information from a smaller set of routinely available physiological measurements.

The growing use of predictive models in healthcare has increased the need to understand how individual patient characteristics influence algorithmic outputs. Explainable artificial intelligence (XAI) addresses this requirement by providing mechanisms for examining the factors underlying model predictions, which is particularly relevant when such predictions are intended to inform clinical decisions. Shapley Additive Explanations (SHAPs) are widely used for this purpose because they provide a principled means of quantifying feature contributions both across a study population and for individual predictions. Their application to mortality modelling has demonstrated how influential physiological characteristics can be identified, and their contributions to predicted risk have been examined in a clinically interpretable manner [6]. Previous biomedical machine learning research has likewise highlighted the value of transparent and reproducible analytical procedures when interpreting physiological and biomedical data [10]. Accordingly, combining predictive modeling with explicit explanation of model behavior may help bridge the gap between algorithmic performance and clinically understandable decision support.

Motivated by these considerations, the present study investigates whether physiological information accumulated during the first 24 h of intensive care can support transparent prediction of subsequent in-hospital mortality. Using MIMIC-IV, we constructed a common experimental setting in which Logistic Regression, Random Forest, XGBoost, LightGBM, and CatBoost were trained and compared using the same study cohort and prediction target. Evaluation extended beyond discrimination alone by examining probability calibration, threshold-dependent classification behavior, and potential clinical utility through calibration curves, confusion matrices, threshold-sensitivity analysis, and decision curve analysis. SHAP was subsequently used to characterize how individual predictors contributed to XGBoost model output at both population and patient levels.

The analysis was deliberately restricted to routinely recorded physiological measurements rather than broad laboratory panels or high-dimensional multimodal inputs. This design examines whether a relatively compact set of physiological observations accumulated across the complete first 24 h of ICU care can provide useful prognostic information while retaining a model structure that can be interrogated through explainability analysis. The proposed framework is intended as an investigational first-24 h mortality-risk assessment approach rather than a substitute for established ICU severity scores or clinical judgment.

The methodological contribution of this study does not arise from introducing a new machine learning algorithm or a new explainability method. Instead, the novelty lies in the integrated evaluation framework, which combines a deliberately compact set of routinely collected physiological variables with a consistent comparison of five established classifiers under the same cohort definition, prediction target, preprocessing strategy, and held-out evaluation protocol. The framework jointly examines discrimination, precision–recall performance, calibration, threshold-dependent classification behavior, decision-curve analysis, and both population-level and patient-level SHAP. This design therefore evaluates not only whether mortality risk can be estimated from bedside physiological measurements alone but also whether the resulting predictions are reliable, interpretable, and potentially useful across clinical decision thresholds without dependence on extensive laboratory panels or high-dimensional multimodal inputs.

The main contributions of this study can be summarized as follows:

- An interpretable approach to in-hospital mortality estimation is constructed from physiological measurements collected across the first 24 h of ICU care.
- Five supervised classifiers—Logistic Regression, Random Forest, XGBoost, LightGBM, and CatBoost—are examined under the same data-processing, training, and testing strategy, allowing direct comparison of their predictive behavior.
- Model assessment incorporates complementary measures of discrimination, minority-class performance, classification quality, and probabilistic reliability, including AUROC, AUPRC, Matthews correlation coefficient, Brier score, calibration curves, confusion matrices, and decision curve analysis.
- SHAP analysis is incorporated at population and individual levels to reveal which physiological characteristics influence model output and how particular features contribute to predictions for representative patients.
- The predictor space is deliberately restricted to routinely recorded early physiological information, providing a relatively compact modeling strategy without requiring extensive laboratory panels or high-dimensional multimodal inputs.
- The complete analysis is performed on MIMIC-IV using a reproducible retrospective experimental design, providing a basis for subsequent testing in independent cohorts and prospective clinical environments.

**Figure 1:**
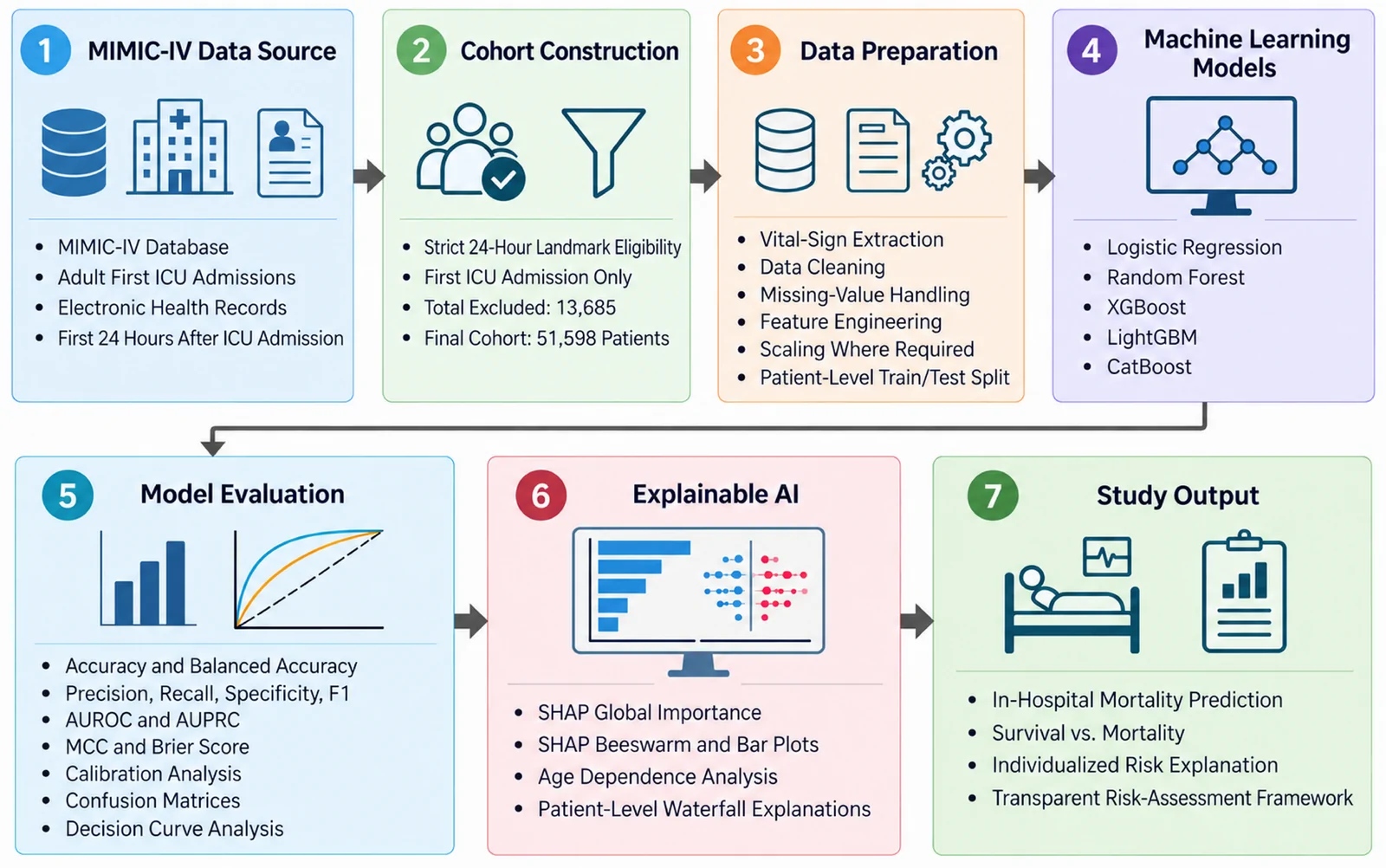
Overall workflow of the proposed explainable machine learning framework for in-hospital mortality prediction using routine physiological variables extracted from the first 24 h after ICU admission.

The analytical pathway is summarized in Figure 1. It proceeds from cohort definition and extraction of physiological measurements within the first 24 h of ICU admission to feature construction, model development and evaluation, and SHAP-based interpretation.

The remainder of this paper is structured as follows. Section 2 reviews related work on machine learning and explainable artificial intelligence for critical care prediction. Section 3 describes the study design, cohort construction, data preprocessing, feature engineering, model development, evaluation criteria, and explainability methodology. Section 4 reports the experimental findings, including predictive performance, calibration, classification outcomes, SHAP-based interpretation, and decision curve analysis. Section 5 discusses the principal findings, clinical implications, strengths, limitations, and directions for future research. Finally, Section 6 summarizes the main conclusions of this study.

## 2. Related Work

The expanding availability of electronic health records (EHRs) has created new opportunities to identify deterioration and adverse outcomes earlier during intensive care. Reliable prognostic information obtained soon after ICU admission may be valuable not only for recognizing vulnerable patients but also for guiding the prioritization of limited critical care resources and supporting individualized management decisions. Considerable differences in resource use and clinical outcomes have been reported across ICUs even after accounting for illness severity, underscoring the value of prognostic approaches that can be evaluated consistently across critically ill populations [11]. Beyond mortality estimation, data-driven methods have been investigated for the earlier recognition of ICU-related complications; for example, machine learning has been used to identify patients at risk of developing ICU-acquired weakness [12]. The broader adoption of artificial intelligence in translational medicine has similarly extended predictive modeling to disease outcomes and patient-centered care across a range of clinical applications [13]. The feasibility of analyzing routinely generated critical care information at scale was demonstrated earlier by Van Poucke et al. through an open platform designed for predictive analysis of ICU data [14]. More recent work by Li et al. applied machine learning to hospital mortality estimation among mechanically ventilated ICU patients, further illustrating the relevance of interpretable risk modeling in critically ill populations [15]. Evidence synthesized by Moor et al. on machine learning for early sepsis prediction likewise points to the growing role of predictive analytics in intensive care while emphasizing that rigorous validation and clinically meaningful interpretation remain important considerations before such systems are incorporated into routine practice [16].

Despite improvements in predictive performance, limited insight into how complex models arrive at their outputs remains an important barrier to their use in clinical settings. When the contribution of individual patient characteristics to a prediction cannot be readily examined, clinicians may have difficulty evaluating the basis of algorithm-generated risk estimates. This concern has increased interest in explainable artificial intelligence (XAI) as a means of making clinical prediction systems more transparent. Vimbi et al. reviewed the growing application of Shapley Additive Explanations (SHAPs) and Local Interpretable Model-Agnostic Explanations (LIME) for interpreting artificial intelligence models used in medical diagnosis and clinical prediction [17]. Among these approaches, SHAP provides a consistent framework for quantifying how individual variables contribute to model output, permitting interpretation across an entire cohort as well as for a specific patient. Nohara et al. demonstrated the application of SHAP to machine learning models developed from real-world hospital data, illustrating how feature-level explanations can provide additional insight into otherwise difficult-to-interpret predictions [18]. Taken together, this body of work indicates that assessment of a clinical prediction model should consider not only its predictive performance but also whether its outputs can be interpreted in a clinically understandable manner.

A separate concern is whether models developed from retrospective clinical data will retain their performance when transferred beyond the environment in which they were trained. Differences in patient characteristics, institutional practices, data collection processes, and changes in care over time can alter the relationship between predictors and outcomes, potentially reducing performance in new settings. Subbaswamy and Saria identified dataset shift as a central challenge for the reliable deployment of artificial intelligence in healthcare and discussed the importance of causal considerations when developing models intended to operate under changing conditions [19]. Addressing a related problem, Wang et al. examined domain-generalization methods designed to improve model performance when applied to previously unseen domains, an issue with particular relevance to multicenter healthcare applications [20]. The importance of careful model development and validation has also been demonstrated in other clinical prediction settings, including the estimation of inadequate bowel preparation among older patients [21]. More recently, Luo et al. developed an interpretable machine learning approach for estimating in-hospital mortality in ICU patients with ischemic stroke, illustrating how prognostic modeling can be combined with explicit examination of the factors contributing to individual predictions [22]. Collectively, these studies reinforce the need to consider transportability, validation, and interpretability alongside predictive performance when developing machine learning systems for critical care.

Although recent studies have substantially advanced machine learning-based prognostic modeling in critical care, several limitations remain. Many published models depend on extensive laboratory investigations, disease-specific biomarkers, or computationally intensive deep learning architectures that may limit implementation in routine clinical practice. In addition, relatively few studies have simultaneously evaluated discrimination, precision–recall performance, calibration, threshold-dependent classification behavior, clinical utility, and model explainability within a unified experimental framework using routinely recorded physiological measurements. Inspired by these constraints, the current study uses a compact set of demographic and routinely obtained physiological variables accumulated during the first 24 h after ICU admission to construct an interpretable machine learning framework for subsequent in-hospital mortality prediction. This study systematically compares five supervised machine learning algorithms and integrates SHAP-based explainability, calibration analysis, decision curve analysis, bootstrap confidence intervals, paired statistical comparisons, and threshold-sensitivity analysis. The resulting framework is intended as a transparent first-24 h ICU risk-assessment approach for further external and prospective evaluation rather than as a real-time prediction system operating at ICU admission.

## 3. Materials and Methods

### 3.1. Study Design

We conducted a retrospective observational study using de-identified electronic health records from the Medical Information Mart for Intensive Care IV (MIMIC-IV) database. The analysis focused on adult ICU patients and examined whether physiological information collected during the first 24 h of ICU admission could support prediction of subsequent in-hospital mortality. Because the predictor representation included summary statistics calculated across the first 24 h observation window, including minimum, maximum, standard deviation, and last recorded values, the present framework should not be interpreted as a real-time prediction made at ICU admission. Rather, it represents mortality-risk estimation based on physiological information accumulated during the first 24 h of ICU care. Model development was accompanied by explainable artificial intelligence (XAI) analysis to characterize the contribution of individual predictors and provide transparent interpretation of mortality-risk estimates.

The proposed framework deliberately focuses on routinely measured vital signs accumulated across the complete first 24 h of ICU care, in contrast to prognostic approaches that rely on extensive laboratory investigations, disease-specific biomarkers, or high-dimensional clinical inputs. Because the predictor representation includes summary features constructed from the complete observation window, the mortality-risk estimates are generated only after the 24 h landmark and should not be interpreted as real-time predictions at ICU admission. Nevertheless, the use of routinely collected physiological variables provides a comparatively compact and interpretable basis for subsequent risk assessment without requiring additional diagnostic tests. Related efforts to develop simplified and clinically practical machine learning models have demonstrated encouraging predictive performance across multiple healthcare applications [23,24,25,26].

The analysis followed five main stages. First, the eligible patient cohort was constructed from MIMIC-IV. Second, routinely documented vital-sign measurements from the first 24 h of each ICU admission were retrieved. Third, the extracted data underwent preprocessing and feature construction before model training. Fourth, several supervised machine learning classifiers were developed and internally evaluated. Finally, Shapley Additive Explanations (SHAPs) were applied to the selected model to examine both population-level feature contributions and patient-specific predictions. Predictive performance was assessed from complementary perspectives, including discrimination, classification performance, probability calibration, and potential clinical usefulness. Accordingly, the evaluation incorporated ROC and precision–recall analyses, confusion matrices, calibration assessment, and decision curve analysis.

Reporting of the study was guided by the Transparent Reporting of a multivariable prediction model for Individual Prognosis Or Diagnosis (TRIPOD) recommendations, with particular attention to transparent description of the study population, predictor processing, model development, validation, and performance assessment [27].

Figure 1 summarizes the study pipeline, from cohort construction and extraction of first-24 h physiological data from MIMIC-IV to feature preparation, model development and evaluation, and SHAP-based interpretation of the selected model.

### 3.2. Data Source

Patient data were obtained from the Medical Information Mart for Intensive Care IV (MIMIC-IV, version 3.1) database, developed through collaboration between the Massachusetts Institute of Technology (MIT) Laboratory for Computational Physiology and Beth Israel Deaconess Medical Center (BIDMC) in Boston, Massachusetts, USA [28]. MIMIC-IV provides de-identified electronic health record data from critically ill patients, encompassing demographic information, hospital and ICU admissions, diagnoses, laboratory measurements, bedside physiological observations, medications, clinical interventions, and patient outcomes. MIMIC-IV contains de-identified clinical data and is distributed through the PhysioNet platform under controlled-access requirements. Access requires credentialed authorization and completion of the required training for research involving human data. Accordingly, the present study involved secondary analysis of de-identified records and did not include direct interaction with patients or any study-specific clinical intervention.

The analysis was restricted to routinely recorded physiological measurements collected during the first 24 h of ICU admission. The selected variables included heart rate (HR), respiratory rate (RR), systolic blood pressure (SBP), diastolic blood pressure (DBP), mean arterial pressure (MAP), and body temperature (TEMP). For each physiological variable, the available measurements were summarized using the mean, standard deviation, minimum, maximum, first recorded value, and last recorded value, thereby representing both the overall level and within-period variation across the complete first 24 h observation window. Shock index was additionally derived as the ratio of heart rate to systolic blood pressure and included as a measure of first-24 h hemodynamic status.

Cohort construction was followed by extraction of physiological observations recorded during the first 24 h of ICU admission. These measurements were aggregated into the predefined patient-level features, checked for data quality, and assembled into the final dataset used for model training and evaluation. The same preparation procedure was applied across all candidate algorithms to maintain a common input representation and permit direct comparison of their predictive performance. Related approaches to structured clinical data preparation have been used in recent machine learning studies involving healthcare prediction tasks [29,30,31].

### 3.3. Study Population and Cohort Selection

Adult ICU admissions recorded in MIMIC-IV were screened according to predefined eligibility criteria. Patients younger than 18 years were excluded, and only the first ICU admission for each patient was retained to avoid correlated observations from repeated ICU stays. Eligibility further required sufficient routinely recorded physiological information during the first 24 h after ICU admission to construct the prespecified predictor features.

Because the predictor representation included summary statistics calculated across the complete first 24 h observation window, a strict 24 h landmark design was applied to reduce temporal information leakage. Patients were required to have a documented ICU duration of at least 24 h, and patients with a documented in-hospital death occurring on or before 24 h after ICU admission were excluded. Among the original analytical cohort of 65,283 patients, 13,502 had a documented ICU stay shorter than 24 h, 12 had missing ICU-duration information, and an additional 171 patients had an ICU duration of at least 24 h but a documented death on or before the 24 h landmark. These mutually exclusive criteria resulted in the exclusion of 13,685 patients.

After application of the 24 h landmark criteria, the revised analytical cohort comprised 51,598 adult patients, including 46,259 survivors and 5339 patients with an in-hospital mortality outcome. Among the mortality cases retained in the revised cohort, 5332 had a documented death timestamp occurring more than 24 h after ICU admission. Seven additional mortality cases had no recorded deathtime; however, each had a documented ICU stay exceeding 24 h and therefore remained eligible for the landmark analysis. No patient with a documented death occurring on or before the 24 h landmark and no patient with a missing or documented ICU duration shorter than 24 h remained in the revised cohort.

### 3.4. Outcome Definition

The study endpoint was in-hospital mortality occurring after the 24 h ICU landmark among patients eligible for prediction at that time point. Outcome status was determined from the documented hospital discharge outcome in MIMIC-IV using the hospital_expire_flag variable. Patients who survived the first 24 h of ICU care but subsequently died during the same hospitalization were assigned to the positive class, whereas patients discharged alive were assigned to the negative class. Patients who died on or before 24 h after ICU admission were not included in the landmark prediction cohort.

The prediction framework was deliberately restricted to physiological information available during the first 24 h of ICU admission. Rather than incorporating laboratory measurements, treatment-related variables, or composite severity scores, the models were developed from routinely recorded vital-sign features and the derived shock index. This restricted predictor set was selected to examine whether first-24 h physiological information alone could provide useful mortality-risk stratification while retaining a comparatively simple and clinically interpretable input structure.

The mortality prediction problem was formulated as a supervised binary classification task. For each patient, the input was represented by a physiological feature vector x

(1) $$x=[x_{1},x_{2},\ldots ,x_{n}],$$

where each element of x corresponds to one of the predictor features derived from the available demographic and first-24 h physiological data. The objective of each classifier was to estimate the conditional probability

(2) P(Y=1∣x),

where the binary outcome was defined as

$$Y=\left\{\begin{array}{cc} 1, & \mathrm{in-hospital}\;\mathrm{death},\\ 0, & \mathrm{survival}\;\mathrm{to}\;\mathrm{hospital}\;\mathrm{discharge}. \end{array}\right.$$

Each machine learning algorithm independently estimated the probability of in-hospital mortality from the same predictor set. Model performance was subsequently examined using complementary measures of discrimination, classification performance, probability calibration, and potential clinical utility. The baseline demographic and physiological characteristics of survivors and non-survivors are summarized in Table 1.

### 3.5. Feature Engineering

Physiological measurements recorded across the complete first 24 h of ICU admission were converted into structured patient-level features for model development. Six routinely monitored variables were included: heart rate (HR), respiratory rate (RR), systolic blood pressure (SBP), diastolic blood pressure (DBP), mean arterial pressure (MAP), and body temperature (TEMP). Restricting the predictor set to these bedside measurements allowed the analysis to examine first-24 h physiological information without incorporating laboratory-derived predictors.

Because vital signs may fluctuate considerably across the first 24 h of ICU care, multiple characteristics of each variable were retained rather than relying on a single measurement. For HR, RR, SBP, DBP, MAP, and TEMP, six summary statistics were calculated from observations recorded across the complete first 24 h window: mean, standard deviation, minimum, maximum, first recorded value, and last recorded value. This representation captured both the overall level and within-period variation of each physiological measurement while maintaining a compact numerical feature set for subsequent machine learning analysis.

The prediction model also included patient demographics. Because these are known predictive markers linked to clinical outcomes in critically sick patients, age and sex were added. Furthermore, a nonlinear age term ($Age^{2}$) was created so that the machine learning algorithms could identify any nonlinear correlations between mortality risk and aging.

To provide an additional measure of hemodynamic status, the shock index (SI) was derived from heart rate and systolic blood pressure as

(3) $$SI=\frac{HR}{SBP},$$

where HR denotes heart rate (beats/min) and SBP denotes systolic blood pressure (mmHg). The shock index was included as a derived physiological feature to capture the relationship between heart rate and arterial blood pressure across the first 24 h observation window.

Following feature construction, the final analytical dataset combined demographic information with the aggregated physiological features derived from bedside measurements recorded during the first 24 h of ICU admission. The baseline demographic and physiological characteristics of the study population are summarized in Table 1.

### 3.6. Data Preprocessing

Prior to model development, the extracted data were processed using a consistent preprocessing pipeline. Each patient was represented by a single feature vector together with the binary in-hospital mortality outcome.

Peripheral oxygen saturation (SpO_2_) was not included in the prespecified predictor set used in this study. The analysis was deliberately restricted to the selected routine physiological variables to maintain a compact and interpretable feature representation. Future work should assess whether inclusion of SpO_2_ and other routinely available bedside measurements provides incremental predictive value beyond the present feature set.

Missing values in continuous predictors were imputed using the median estimated from the training data. The numerical predictors were subsequently standardized using parameters estimated from the training set. For the categorical sex variable, missing observations were replaced with the most frequent category and the resulting variable was one-hot encoded. These preprocessing operations were incorporated into the modeling pipeline so that preprocessing parameters were estimated from the training data and subsequently applied to the held-out test data, thereby reducing the risk of information leakage.

Missingness was quantified in the revised analytical cohort before imputation. Missing values were minimal for heart-rate and respiratory-rate features but were more frequent for blood-pressure and temperature summaries. Specifically, 5 values (0.01%) were missing for heart-rate standard deviation, and respiratory-rate features had 114–146 missing observations (0.22–0.28%). Systolic blood pressure summary features had 5629 missing observations (10.91%) for most statistics and 7081 (13.72%) for the standard deviation. Corresponding missingness ranged from 5635 to 7087 observations (10.92–13.74%) for diastolic blood pressure and from 5647 to 7092 observations (10.94–13.74%) for mean arterial pressure. Temperature features contained 1544 missing observations (2.99%) for most summary statistics and 2109 (4.09%) for the standard deviation, while the derived mean shock index contained 5630 missing values (10.91%). These missing predictor values were retained and handled within the training data preprocessing pipeline using median imputation rather than excluding additional patients.

Physiological observations were restricted to the predefined MIMIC-IV item identifiers and to measurements with a numeric valuenum recorded within the first 24 h after ICU admission. Temperature measurements recorded in degrees Fahrenheit were converted to degrees Celsius before feature construction. No additional post hoc physiological range-based outlier filtering was applied to the extracted vital sign values; because rare extreme recorded values were present in some physiological distributions, baseline continuous characteristics were summarized using median and interquartile range rather than mean and standard deviation. Summary features were subsequently constructed from the available observations for each patient, and all preprocessing and imputation parameters used for model development were estimated from the training data only.

The final analytical cohort was divided into training and testing sets using a stratified 80:20 patient-level split with a fixed random seed of 42. This resulted in 41,278 patients in the training set and 10,320 patients in the held-out test set. The training set included 37,007 survivors and 4271 patients with an in-hospital mortality outcome, while the test set included 9252 survivors and 1068 patients with an in-hospital mortality outcome, preserving a mortality prevalence of approximately 10.35% in both subsets. Model fitting, preprocessing parameter estimation, hyperparameter optimization, and internal cross-validation were conducted using the training data, whereas the held-out test set was reserved for final performance evaluation.

Given the imbalance between survivors and non-survivors, class weighting was incorporated for selected classifiers rather than applying synthetic oversampling or undersampling. Logistic Regression used balanced class weights, while Random Forest used balanced subsample weighting during tree construction. XGBoost, LightGBM, and CatBoost were trained without explicit class weighting in the final configurations, and no synthetic resampling procedure such as SMOTE was applied. Class weighting was applied in an algorithm-specific manner rather than imposed uniformly across all classifiers. The native balanced weighting options were retained for Logistic Regression and Random Forest to increase the contribution of the minority mortality class during model fitting. In contrast, XGBoost, LightGBM, and CatBoost were evaluated in their final configurations without explicit class weighting, allowing their native boosting procedures to learn from the original class distribution. This strategy also avoided introducing synthetic samples and enabled comparison of the classifiers under their selected modeling configurations. Model performance was therefore evaluated using several metrics that are informative under class imbalance, including balanced accuracy, AUPRC, F1-score, and the Matthews correlation coefficient, in addition to conventional accuracy and AUROC.

This preprocessing strategy provided a consistent framework for comparing the five supervised machine learning algorithms while maintaining separation between model development and final evaluation. Similar structured preprocessing and model-development strategies have been adopted in recent clinical machine learning studies to support reproducible and systematic evaluation of predictive models [25,26,29,30,31].

### 3.7. Machine Learning Model Development

Five supervised machine learning algorithms were evaluated for in-hospital mortality prediction: Logistic Regression (LR), Random Forest (RF), Extreme Gradient Boosting (XGBoost), Light Gradient Boosting Machine (LightGBM), and Categorical Boosting (CatBoost). These models were selected to represent different modeling strategies, including linear classification, bagging-based ensemble learning, and gradient boosting. Similar approaches have been applied in recent clinical prediction studies using structured healthcare data [29,30,31].

After preprocessing, the resulting feature matrix was used for model development under a common experimental protocol. Hyperparameter optimization and internal validation were restricted to the training data, and the held-out test set was used only after model development to obtain an independent comparison of predictive performance across the five classifiers.

#### 3.7.1. Logistic Regression

The baseline statistical learning model was Logistic Regression, which was chosen due to its widespread clinical application in binary outcome prediction, computational efficiency, and simplicity. The logistic function is employed to estimate the likelihood of in-hospital mortality in the model.

(4) $$P\left(Y=1|x\right)=\frac{1}{1+\exp^{-(\beta_{0}+\sum_{i=1}^{n}\beta_{i}x_{i})}},$$

where x denotes the feature vector, $\beta_{0}$ is the intercept, and $\beta_{i}$ represents the regression coefficient associated with the $i^{th}$ predictor.

#### 3.7.2. Random Forest

Random Forest is an ensemble learning algorithm that employs random feature selection and bootstrap sampling to generate numerous decision trees. The model’s robustness against overfitting is enhanced, and the variance is reduced, by obtaining final predictions thru majority voting across all individual trees.

#### 3.7.3. Extreme Gradient Boosting (XGBoost)

XGBoost implements gradient-boosted decision trees in a sequential ensemble, with each new tree fitted to improve errors remaining from the preceding boosting steps. Its training objective combines the predictive loss with regularization of model complexity, allowing nonlinear and interaction effects to be learned while limiting excessive model complexity.

#### 3.7.4. Light Gradient Boosting Machine (LightGBM)

In order to facilitate efficient learning from high-dimensional structured data while maintaining a relatively low computational cost, LightGBM implements a histogram-based gradient boosting strategy in conjunction with leaf-wise tree growth. LightGBM has gained popularity for large-scale clinical prediction assignments due to its strong predictive capability and computational efficiency.

#### 3.7.5. Categorical Boosting (CatBoost)

CatBoost is a gradient boosting method that uses ordered boosting to reduce prediction bias during sequential tree construction. Although the algorithm provides dedicated mechanisms for handling categorical predictors, it can also be applied to structured numerical data such as the feature representation used in this study.

Hyperparameter selection for all five classifiers was performed within the training set using five-fold cross-validation, with AUROC as the optimization criterion. The final configurations were: Logistic Regression, C=10 with L2 regularization and the lbfgs solver; Random Forest, 200 trees, maximum depth of 20, minimum leaf size of 5, and sqrt feature sampling; XGBoost, 300 estimators, learning rate of 0.05, maximum depth of 5, subsample ratio of 0.8, and column-sampling ratio of 0.8; LightGBM, 200 estimators, learning rate of 0.05, maximum depth of 8, and 31 leaves; and CatBoost, 500 iterations, depth of 6, and learning rate of 0.05. After hyperparameter selection, each fitted model was applied to the held-out test set under the same evaluation protocol.

### 3.8. Model Evaluation and Explainability

Model performance was evaluated exclusively on the held-out test set, which was not involved in model fitting or hyperparameter selection. Evaluation was conducted across several complementary dimensions, including discrimination, classification performance, probability calibration, and potential clinical utility. Multiple metrics were therefore considered rather than relying on a single performance measure, consistent with current guidance for evaluating machine learning models in biomedical applications [32].

Overall classification accuracy was calculated as

(5) $$Accuracy=\frac{TP+TN}{TP+TN+FP+FN},$$

where TP, TN, FP, and FN denote true positives, true negatives, false positives, and false negatives, respectively.

Because mortality prediction datasets are inherently imbalanced, balanced accuracy was additionally reported as

(6) $$Balanced\;Accuracy=\frac{Sensitivity+Specificity}{2},$$

where

(7) $$Sensitivity=\frac{TP}{TP+FN},$$

and

(8) $$Specificity=\frac{TN}{TN+FP}.$$

Precision and recall were computed as

(9) $$Precision=\frac{TP}{TP+FP},$$

(10) $$Recall=\frac{TP}{TP+FN},$$

while the harmonic mean of precision and recall was expressed as

(11) $$F1=2\left(\frac{Precision\times Recall}{Precision+Recall}\right).$$

To evaluate the quality of binary classification under class imbalance, the Matthews correlation coefficient (MCC) was also calculated as

(12) $$MCC=\frac{TP\times TN-FP\times FN}{\sqrt{\left(TP+FP)(TP+FN)(TN+FP)(TN+FN\right)}}.$$

Discriminative performance was quantified using the area under the receiver operating characteristic curve (AUROC) and the area under the precision–recall curve (AUPRC). AUROC characterizes the model’s ability to distinguish survivors from non-survivors across classification thresholds, whereas AUPRC focuses on the relationship between precision and recall and is particularly informative when evaluating the less frequent mortality class.

Calibration was examined using calibration curves constructed from 15 equal-width predicted-probability intervals and the Brier score. The Brier score quantifies the average squared difference between the predicted probability of mortality and the observed binary outcome:

(13) $$Brier=\frac{1}{N}\sum_{i=1}^{N}{\left(p_{i}-y_{i}\right)}^{2},$$

where $p_{i}$ denotes the predicted probability of mortality for patient *i*, and $y_{i}$ represents the corresponding observed outcome.

Decision Curve Analysis (DCA) was used to estimate the net clinical benefit over a range of threshold probabilities in order to further evaluate possible clinical value. DCA provides insight into the usefulness of model-assisted clinical decision-making by assessing whether the application of a prediction model offers more clinical benefit than approaches that treat all patients or none at all.

**Figure 2:**
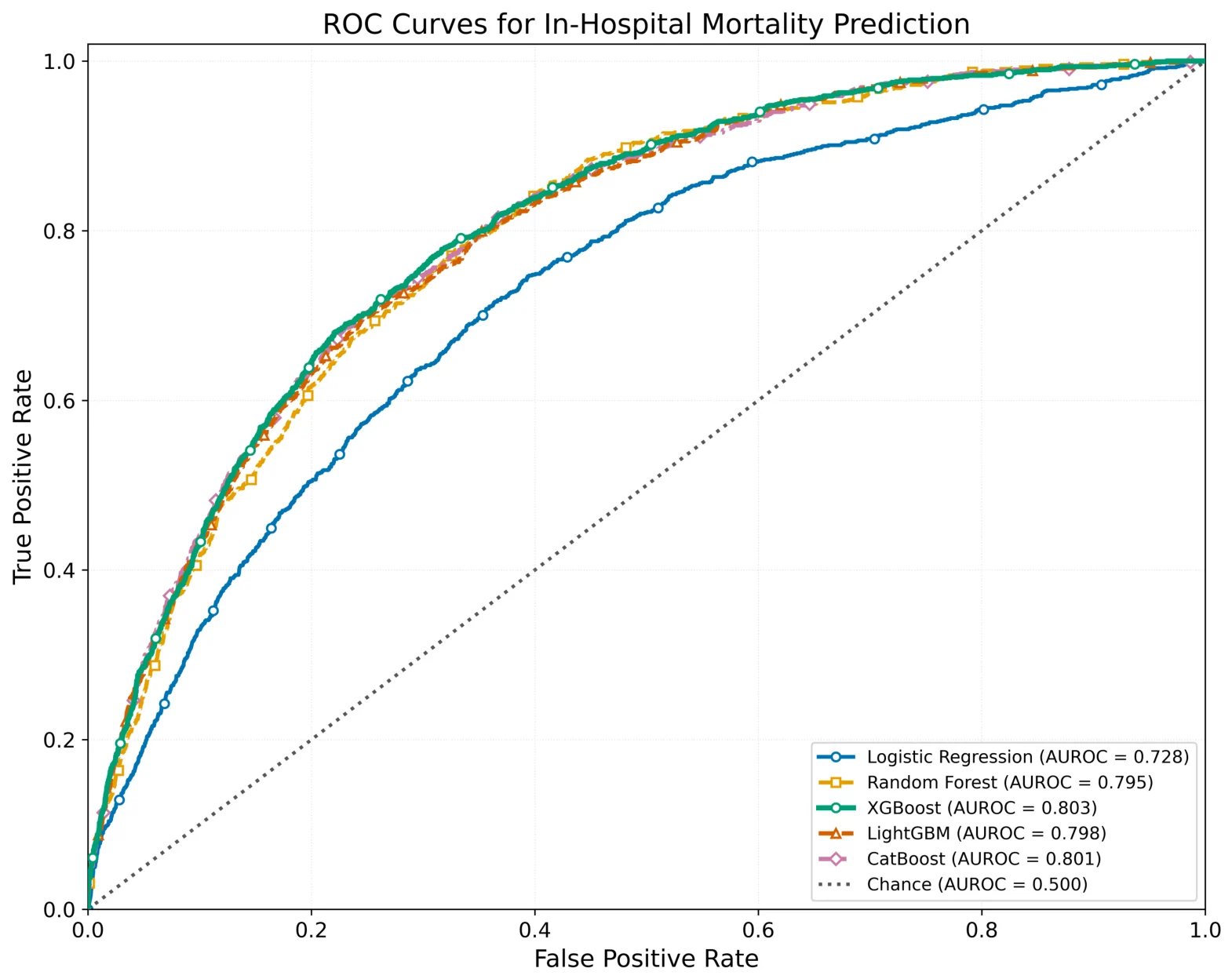
Receiver operating characteristic (ROC) curves comparing the discrimination performance of the five evaluated machine learning models in the revised 24 h landmark cohort. XGBoost achieved an AUROC of 0.803, followed by CatBoost (0.801), LightGBM (0.798), Random Forest (0.795), and Logistic Regression (0.728). The diagonal reference line represents chance-level discrimination (AUROC = 0.500).

**Figure 3:**
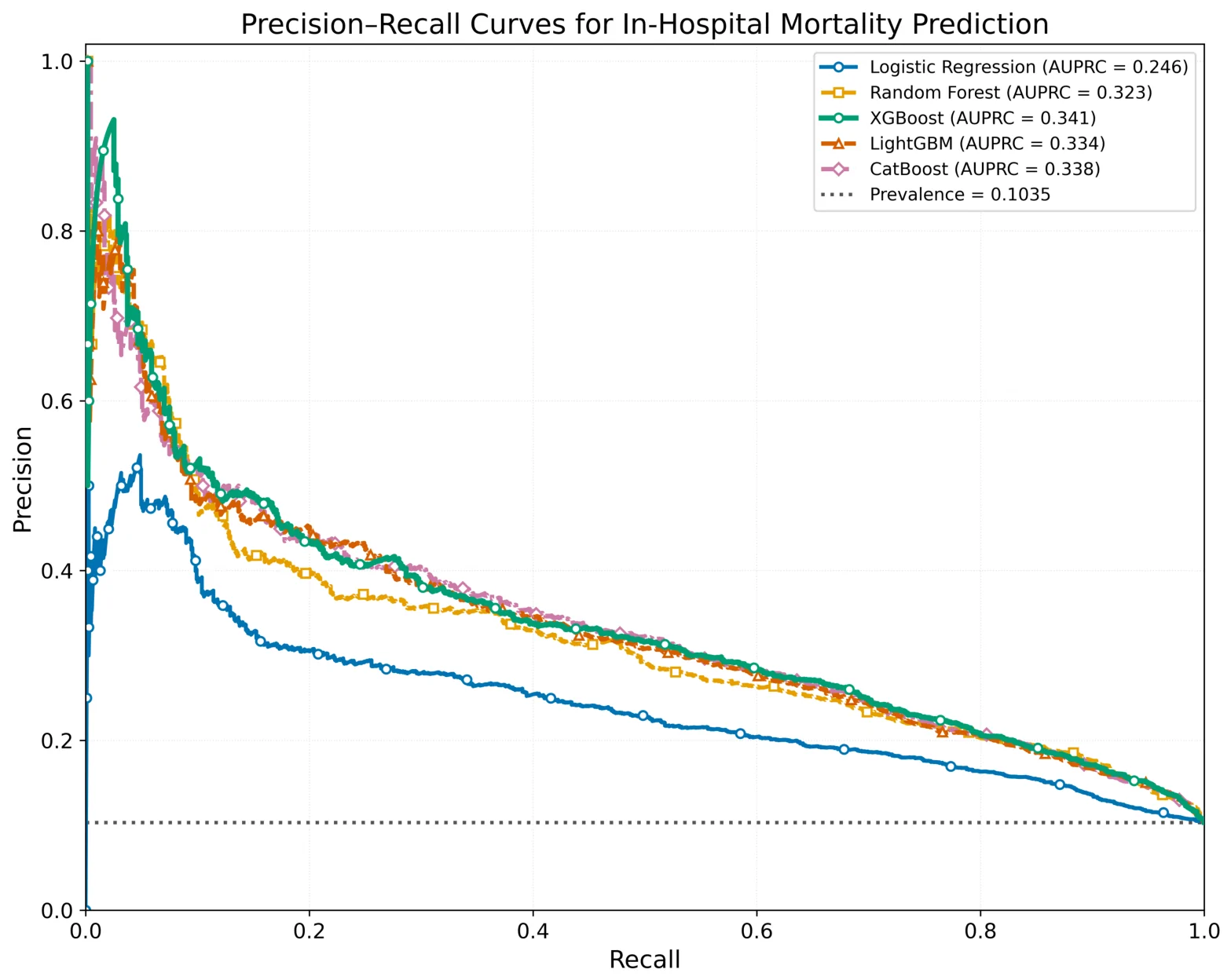
Precision–recall curves of the five evaluated machine learning models for predicting in-hospital mortality in the revised 24 h landmark cohort. XGBoost achieved an AUPRC of 0.341, followed by CatBoost (0.338), LightGBM (0.334), Random Forest (0.323), and Logistic Regression (0.246). The horizontal reference line represents the mortality prevalence in the held-out test cohort (0.1035).

**Figure 4:**
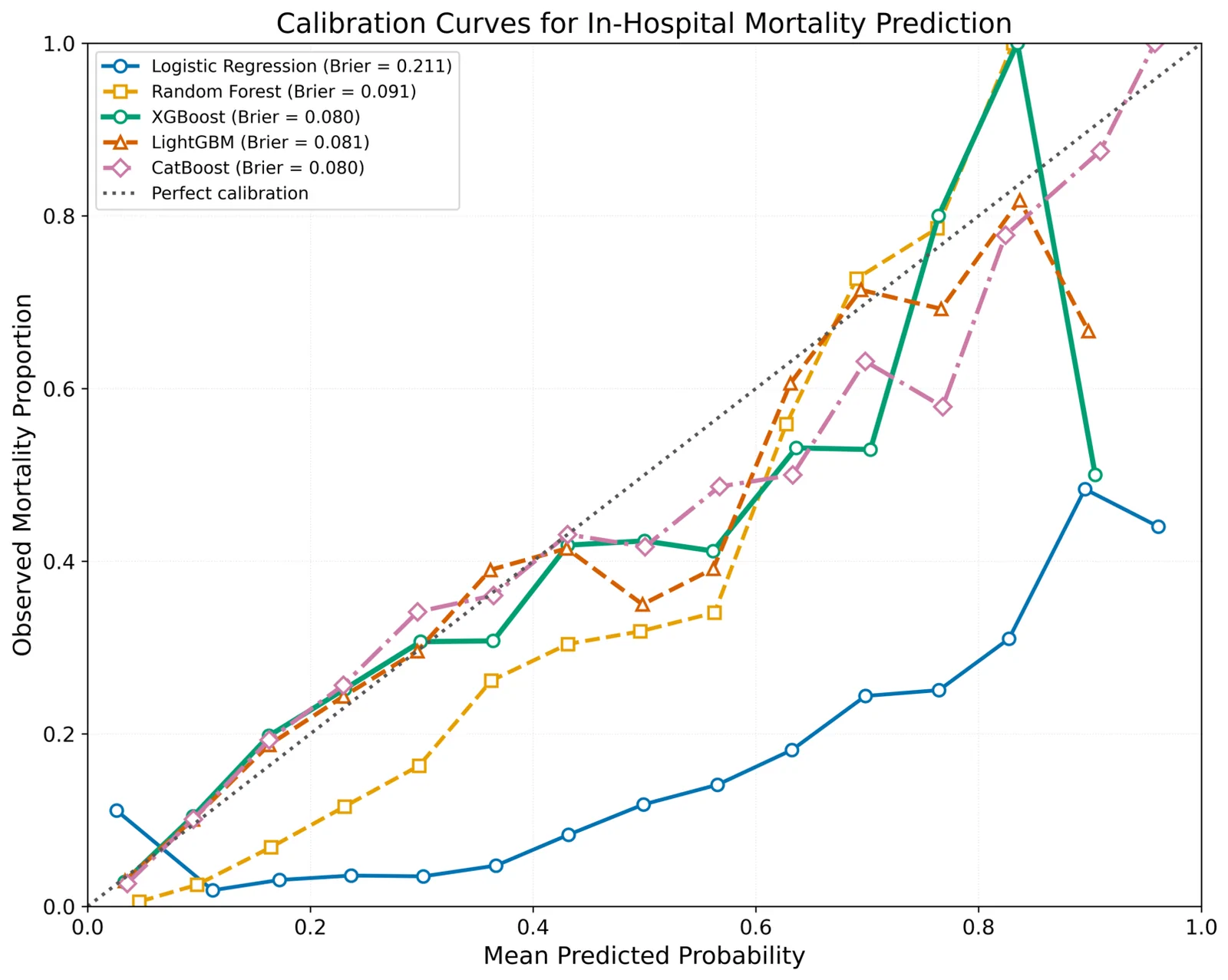
Calibration curves of the five evaluated machine learning models for predicting in-hospital mortality in the revised 24 h landmark cohort, constructed using 15 equal-width predicted-probability intervals. The diagonal reference line represents perfect calibration. XGBoost and CatBoost achieved rounded Brier scores of 0.080, followed by LightGBM (0.081), Random Forest (0.091), and Logistic Regression (0.211). Lower Brier scores indicate lower overall probabilistic prediction error.

**Figure 5:**
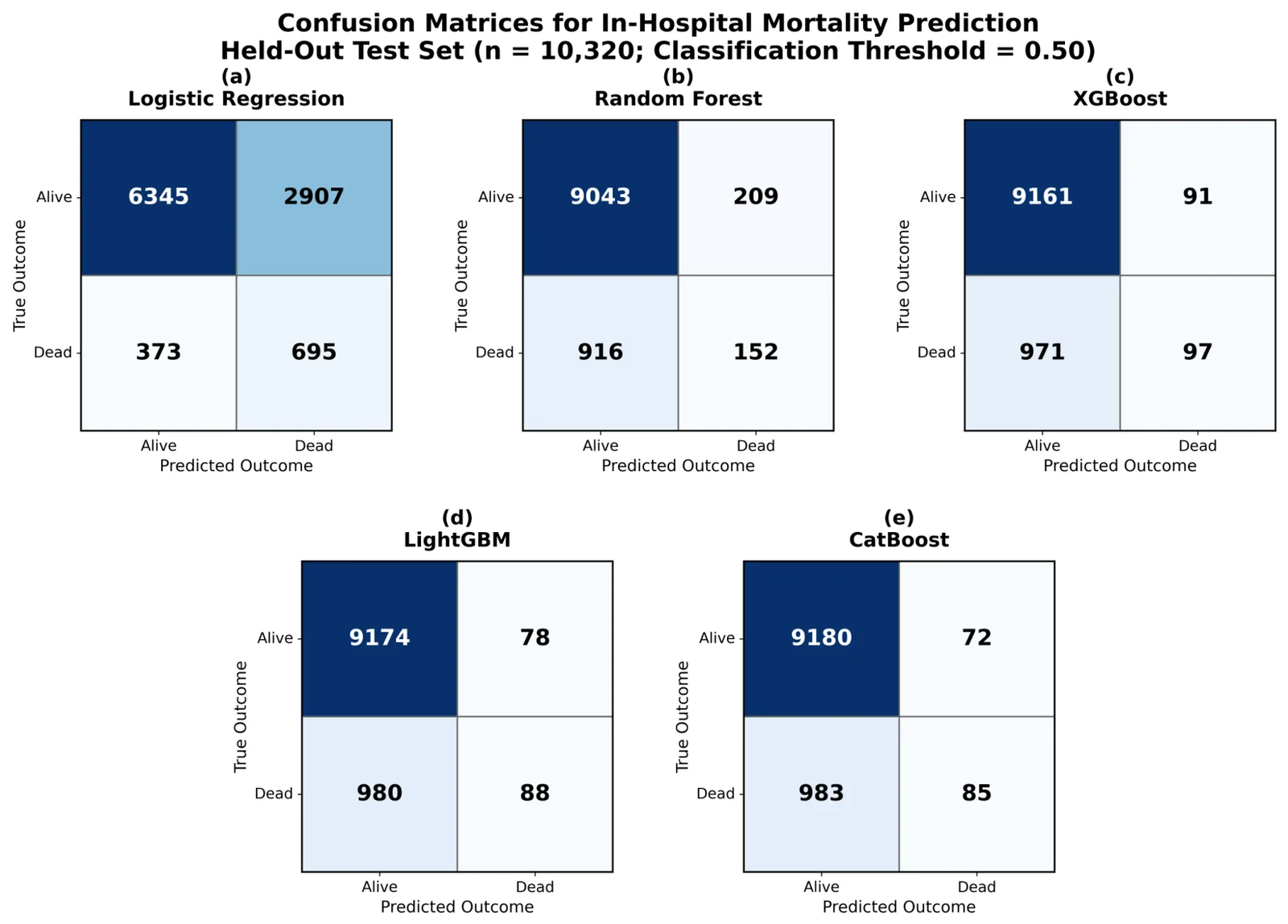
Confusion matrices of the five evaluated machine learning models on the held-out internal test set (n=10,320) using the default classification threshold of 0.50. Rows represent true outcomes and columns represent predicted outcomes for (**a**) Logistic Regression, (**b**) Random Forest, (**c**) XGBoost, (**d**) LightGBM, and (**e**) CatBoost. Logistic Regression identified the largest number of mortality cases but generated substantially more false-positive classifications, whereas the tree-based ensemble models maintained higher specificity.

Model performance is illustrated using ROC curves (Figure 2), precision–recall curves (Figure 3), calibration curves (Figure 4), and confusion matrices (Figure 5). The corresponding quantitative performance metrics for all five classifiers are reported in Table 2.

For threshold-dependent classification measures, including accuracy, balanced accuracy, precision, recall, specificity, F1-score, Matthews correlation coefficient, and confusion-matrix counts, predicted probabilities were converted to binary class labels using the conventional probability threshold of 0.50. AUROC and AUPRC were evaluated independently of a single classification threshold, while the Brier score was calculated directly from predicted probabilities. In addition, threshold-sensitivity analysis was performed for the representative XGBoost model across probability thresholds from 0.05 to 0.50 to characterize the trade-off between mortality detection and false-positive classifications. The 0.50 threshold was retained as the primary reporting threshold and was not optimized using the held-out test set.

To quantify the uncertainty of the principal predictive-performance estimates, 95% confidence intervals were calculated for AUROC, AUPRC, and Brier score using nonparametric bootstrap resampling of the held-out test set. A total of 2000 bootstrap samples were generated with replacement, and the 2.5th and 97.5th percentiles of the bootstrap distributions were used to define the corresponding 95% confidence intervals.

Pairwise statistical comparisons between classifiers were performed using paired bootstrap resampling of the same held-out test observations. For each model pair, differences in AUROC, AUPRC, and Brier score were calculated across 2000 bootstrap resamples. Two-sided bootstrap *p*-values and 95% confidence intervals for the paired differences were used to assess whether observed performance differences were statistically distinguishable.

### 3.9. Model Explainability Using Shapley Additive Explanations (SHAPs)

XGBoost was selected as the representative model for Shapley Additive Explanations (SHAPs) because it achieved the numerically highest AUROC and AUPRC and the lowest unrounded Brier score in the revised analysis. Paired bootstrap comparisons indicated a significantly higher AUROC for XGBoost than for LightGBM but no significant difference from CatBoost; its AUPRC and Brier score did not differ significantly from either LightGBM or CatBoost. Therefore, selection of XGBoost for explainability analysis should not be interpreted as evidence of consistent statistical superiority across all evaluated metrics. SHAP is a game-theoretic explainability approach that estimates each feature’s marginal contribution to model output for both population-level and individual-patient interpretation.

For a prediction model f(x), the SHAP explanation is expressed as

(14) $$f\left(x\right)=\phi_{0}+\sum_{i=1}^{M}\phi_{i},$$

where $\phi_{0}$ represents the expected model prediction over the training population, and $\phi_{i}$ denotes the contribution of the $i^{th}$ feature toward the prediction of an individual patient.

In order to measure overall feature relevance throughout the study population, global model interpretation was carried out using SHAP summary plots and mean absolute SHAP values. In order to facilitate the clinical interpretation of nonlinear feature effects, feature dependence plots were further created to examine the link between significant physiological variables and their associated SHAP contributions.

SHAP waterfall plots were created for representative ICU patients to illustrate interpretability at the patient level. These visualizations show how individual physiological variables contributed positively or negatively to the XGBoost model output for specific predictions.

**Figure 6:**
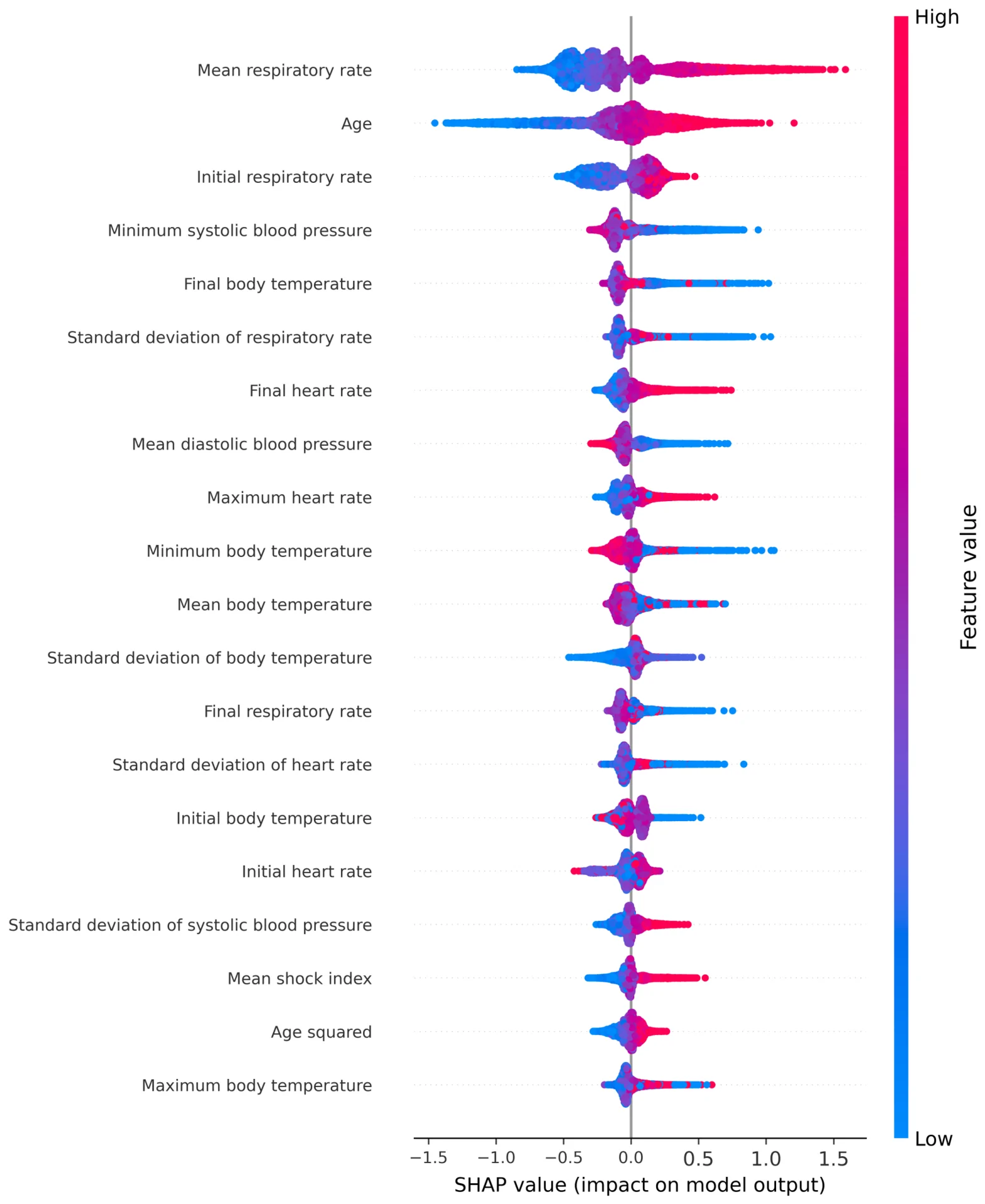
SHAP summary (beeswarm) plot illustrating the global importance and distribution of feature contributions in the XGBoost model. Each point represents one patient, while color indicates the corresponding feature value (blue: low, red: high). Features are ranked according to their mean absolute SHAP values.

**Figure 7:**
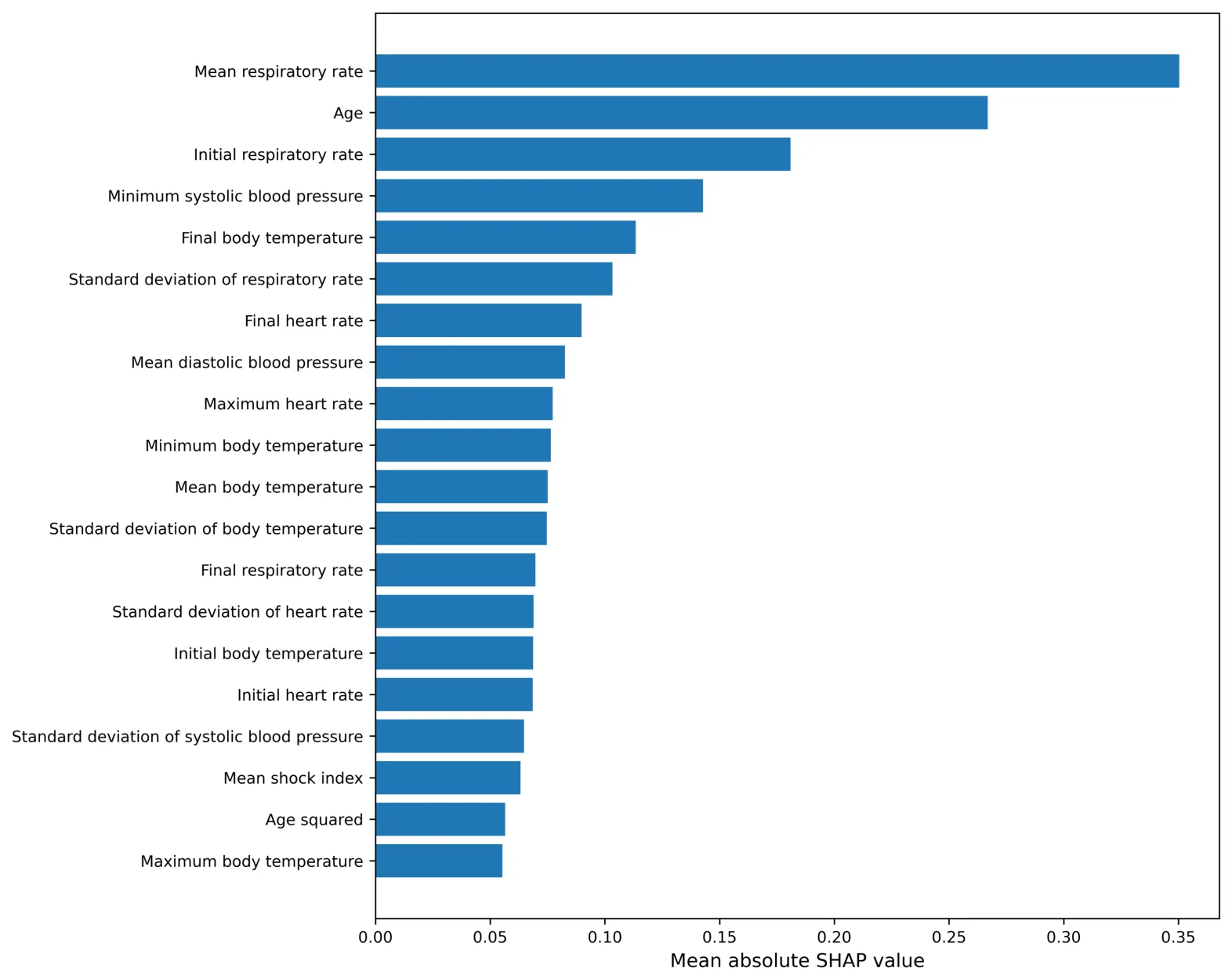
Global feature importance based on mean absolute SHAP values for the final XGBoost model. Features are ranked according to their average absolute contribution to mortality prediction across the held-out test cohort. Mean respiratory rate showed the largest mean absolute SHAP value, followed by age, initial respiratory rate, minimum systolic blood pressure, and final body temperature.

**Figure 8:**
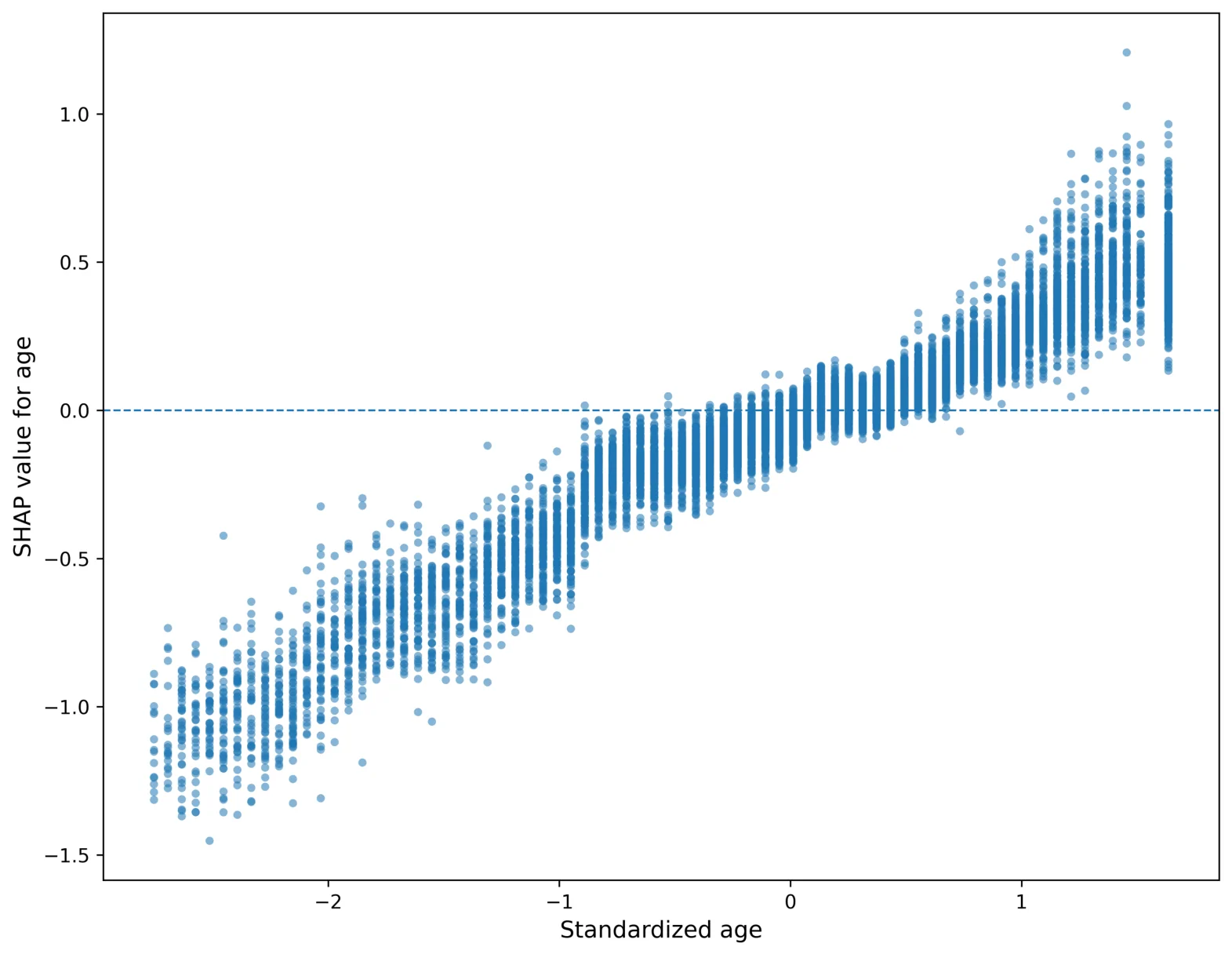
SHAP dependence plot for age in the final XGBoost model. The horizontal axis represents standardized age, and the vertical axis represents the SHAP contribution of age to the model output. Increasing standardized age values were generally associated with progressively larger positive SHAP contributions, indicating that higher age increasingly shifted the model output toward the mortality class.

**Figure 9:**
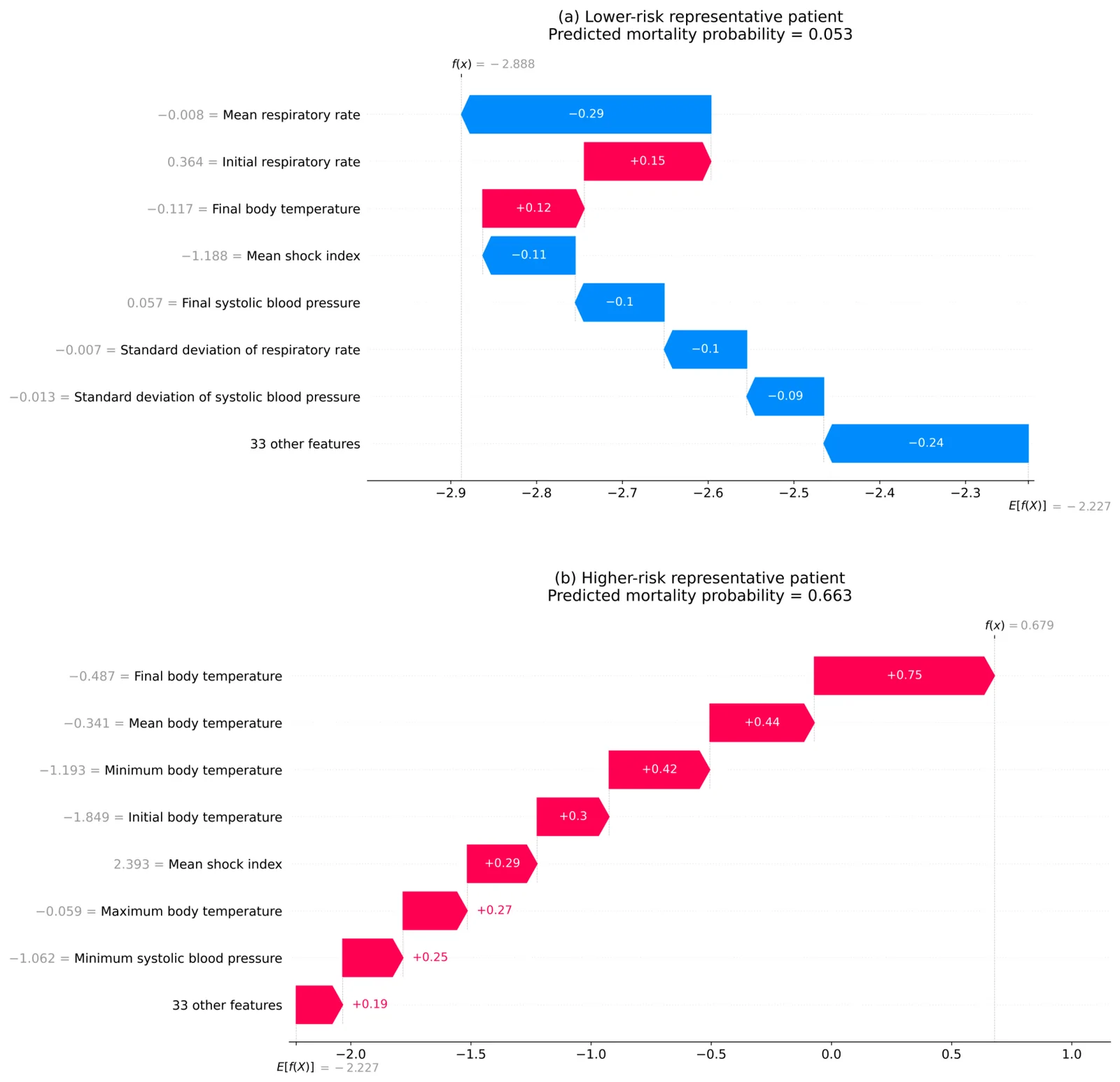
Patient-level SHAP waterfall explanations for two representative correctly classified patients from the held-out test set using the final XGBoost model. Panel (**a**) shows a lower-risk survivor with a predicted mortality probability of 0.053, whereas panel (**b**) shows a higher-risk patient with an in-hospital mortality outcome and a predicted mortality probability of 0.663. Red features increase the XGBoost model output toward the mortality class, while blue features decrease the model output. The displayed feature values correspond to the preprocessed model inputs used for prediction.

The SHAP-based model interpretation is presented through the SHAP beeswarm plot (Figure 6), SHAP bar plot (Figure 7), dependence analysis (Figure 8), and patient-level waterfall explanations (Figure 9). The twenty most influential predictors identified by mean absolute SHAP values are summarized in Table 3.

### 3.10. Statistical Analysis

All data preparation, feature extraction, machine learning model creation, statistical evaluations, and visual representations were conducted utilizing Python 3.11.7 (Python Software Foundation, Wilmington, DE, USA). Data processing was performed via the Pandas and NumPy libraries, and machine learning methods were executed with Scikit-learn alongside the XGBoost, LightGBM, and CatBoost frameworks. SHAP evaluations were conducted with the SHAP Python library.

Continuous variables were summarized as median and interquartile range (IQR), whereas categorical variables were reported as counts and percentages. Differences in continuous baseline characteristics between survivors and patients with an in-hospital mortality outcome were assessed using the two-sided Mann–Whitney U test. Differences in sex distribution were evaluated using the chi-square test of independence. Missing observations were excluded from the corresponding variable-specific descriptive summaries and statistical comparisons. All statistical tests were two-sided, and a *p*-value < 0.05 was considered statistically significant.

## 4. Results

### 4.1. Baseline Characteristics of the Study Population

Table 1 summarizes the baseline characteristics of the revised 24 h landmark cohort. The final analytical cohort comprised 51,598 patients, including 46,259 survivors and 5339 patients with an in-hospital mortality outcome, corresponding to a mortality prevalence of 10.35%. Continuous variables are reported as median [interquartile range (IQR)] because the distributions contained rare extreme recorded values and were therefore summarized using robust descriptive statistics. Patients with an in-hospital mortality outcome were older than survivors (71.00 [60.00–81.00] vs. 65.00 [53.00–76.00] years; p<0.001) and had higher first-24-hour mean heart rate (89.00 [76.69–101.89] vs. 82.29 [73.15–93.11] beats/min; p<0.001), respiratory rate (20.59 [17.76–24.04] vs. 18.21 [16.27–20.68] breaths/min; *p* < 0.001), and shock index (0.79 [0.65–0.95] vs. 0.71 [0.60–0.83]; p<0.001). Conversely, systolic blood pressure, diastolic blood pressure, and mean arterial pressure were lower among patients with an in-hospital mortality outcome than among survivors (all p<0.001). Body temperature also differed statistically between the groups (p<0.001), although the absolute difference in median values was small. Female patients represented 43.0% of the overall cohort, 42.7% of survivors, and 45.8% of patients with an in-hospital mortality outcome (p<0.001).

### 4.2. Comparative Performance of Machine Learning Models

Table 2 presents the performance of the five machine learning models on the held-out test set derived from the revised 24 h landmark cohort. The test set included 10,320 patients, comprising 9252 survivors and 1068 patients with an in-hospital mortality outcome, corresponding to a mortality prevalence of 10.35%. Threshold-dependent performance measures were calculated using the default classification threshold of 0.50.

XGBoost achieved an accuracy of 0.897, balanced accuracy of 0.540, precision of 0.516, recall of 0.091, specificity of 0.990, F1-score of 0.154, AUROC of 0.803, AUPRC of 0.341, MCC of 0.184, and a Brier score of 0.080. LightGBM achieved an accuracy of 0.897, balanced accuracy of 0.537, precision of 0.530, recall of 0.082, specificity of 0.992, F1-score of 0.143, AUROC of 0.798, AUPRC of 0.334, MCC of 0.179, and a Brier score of 0.081.

CatBoost achieved an accuracy of 0.898, balanced accuracy of 0.536, precision of 0.541, recall of 0.080, specificity of 0.992, F1-score of 0.139, AUROC of 0.801, AUPRC of 0.338, MCC of 0.179, and a Brier score of 0.080. Random Forest achieved an accuracy of 0.891, balanced accuracy of 0.560, precision of 0.421, recall of 0.142, specificity of 0.977, F1-score of 0.213, AUROC of 0.795, AUPRC of 0.323, MCC of 0.198, and a Brier score of 0.091.

Logistic Regression showed a markedly different classification profile. It achieved an accuracy of 0.682, balanced accuracy of 0.668, precision of 0.193, recall of 0.651, specificity of 0.686, F1-score of 0.298, AUROC of 0.728, AUPRC of 0.246, MCC of 0.215, and a Brier score of 0.211. Thus, Logistic Regression identified substantially more mortality cases at the default threshold than the tree-based models, but this higher sensitivity was accompanied by considerably lower precision and specificity.

Considering the performance measures collectively, XGBoost achieved the numerically highest AUROC and AUPRC and the lowest unrounded Brier score. However, paired bootstrap comparisons did not establish consistent superiority over both LightGBM and CatBoost across all three metrics. XGBoost was therefore used as the representative model for subsequent SHAP-based interpretability analyses based on its numerical performance rather than evidence of universal statistical superiority.

Bootstrap uncertainty analysis showed that XGBoost achieved an AUROC of 0.803 (95% CI: 0.790–0.815), an AUPRC of 0.341 (95% CI: 0.313–0.369), and a Brier score of 0.080 (95% CI: 0.076–0.084). LightGBM yielded an AUROC of 0.798 (95% CI: 0.785–0.811), an AUPRC of 0.334 (95% CI: 0.306–0.361), and a Brier score of 0.081 (95% CI: 0.077–0.084), while CatBoost achieved an AUROC of 0.801 (95% CI: 0.788–0.815), an AUPRC of 0.338 (95% CI: 0.312–0.368), and a Brier score of 0.080 (95% CI: 0.076–0.084). Random Forest achieved an AUROC of 0.795 (95% CI: 0.782–0.808), an AUPRC of 0.323 (95% CI: 0.295–0.351), and a Brier score of 0.091 (95% CI: 0.088–0.094), whereas Logistic Regression achieved an AUROC of 0.728 (95% CI: 0.712–0.745), an AUPRC of 0.246 (95% CI: 0.223–0.272), and a Brier score of 0.211 (95% CI: 0.208–0.214).

Paired bootstrap comparisons showed that XGBoost significantly outperformed Logistic Regression for AUROC (difference = 0.0744, 95% CI: 0.0620–0.0871, p=0.001), AUPRC (difference = 0.0957, 95% CI: 0.0728–0.1181, p=0.001), and Brier score (difference = −0.1304, 95% CI: −0.1351 to −0.1256, p=0.001). XGBoost also outperformed Random Forest for AUROC (difference = 0.0074, 95% CI: 0.0018–0.0130, p=0.011), AUPRC (difference = 0.0187, 95% CI: 0.0031–0.0344, p=0.021), and Brier score (difference = −0.0106, 95% CI: −0.0122 to −0.0090, p=0.001). Compared with LightGBM, XGBoost had a significantly higher AUROC (difference = 0.0047, 95% CI: 0.0012–0.0082, p=0.012), but differences in AUPRC (p=0.106) and Brier score (p=0.296) were not statistically significant. Differences between XGBoost and CatBoost were not statistically significant for AUROC (p=0.458), AUPRC (p=0.599), or Brier score (p=0.741).

At the default probability threshold of 0.50, XGBoost showed high specificity (0.990) but low sensitivity, identifying 9.1% of mortality cases (recall = 0.091; 97 true positives and 971 false negatives). Threshold-sensitivity analysis demonstrated that this operating point strongly favored specificity over mortality-case detection. Lowering the threshold to 0.20 increased recall to 0.434 while maintaining specificity of 0.899 and precision of 0.331. At a threshold of 0.10, recall increased to 0.728, with specificity of 0.727 and precision of 0.235. These findings indicate that the default 0.50 threshold was not an optimized clinical operating point and that the balance between missed mortality cases and false-positive alerts depended strongly on the selected decision threshold.

### 4.3. Receiver Operating Characteristic Curve Analysis

Receiver operating characteristic (ROC) analysis was performed to evaluate the discriminative ability of the five machine learning algorithms for predicting in-hospital mortality in the revised 24 h landmark cohort. The ROC curves for all evaluated models are presented in Figure 2.

XGBoost achieved the highest AUROC of 0.803, followed by CatBoost (0.801), LightGBM (0.798), Random Forest (0.795), and Logistic Regression (0.728). Bootstrap uncertainty analysis yielded an AUROC of 0.803 (95% CI: 0.790–0.815) for XGBoost, 0.801 (95% CI: 0.788–0.815) for CatBoost, 0.798 (95% CI: 0.785–0.811) for LightGBM, 0.795 (95% CI: 0.782–0.808) for Random Forest, and 0.728 (95% CI: 0.712–0.745) for Logistic Regression.

Paired bootstrap comparisons showed that XGBoost had a significantly higher AUROC than LightGBM (difference = 0.0047, 95% CI: 0.0012–0.0082, p=0.012), whereas its AUROC did not differ significantly from that of CatBoost (difference = 0.0016, 95% CI: −0.0027–0.0059, p=0.458). XGBoost was used as the representative model for subsequent SHAP-based interpretability analyses because it achieved the highest numerical AUROC and AUPRC and the lowest unrounded Brier score, rather than because it was consistently statistically superior to the other gradient-boosting models across all evaluated metrics.

### 4.4. Precision–Recall Curve Analysis

Given the class imbalance in the revised 24-h landmark cohort, with mortality representing 10.35% of the held-out test set, precision–recall (PR) curve analysis was performed to provide an additional assessment of model performance for the minority mortality class. Unlike ROC analysis, PR curves emphasize the balance between precision and recall and are particularly informative when the positive outcome is relatively infrequent.

Figure 3 compares the precision–recall profiles of the five classifiers. XGBoost achieved the numerically highest AUPRC of 0.341, followed by CatBoost (0.338), LightGBM (0.334), Random Forest (0.323), and Logistic Regression (0.246).

Paired bootstrap comparisons showed that the AUPRC differences between XGBoost and LightGBM (p=0.106) and between XGBoost and CatBoost (p=0.599) were not statistically significant. However, XGBoost achieved a significantly higher AUPRC than Random Forest (difference = 0.0187, 95% CI: 0.0031–0.0344, p=0.021) and Logistic Regression (difference = 0.0957, 95% CI: 0.0728–0.1181, p=0.001).

Although Logistic Regression achieved substantially higher recall at the default classification threshold of 0.50, this occurred with markedly lower precision. In contrast, the gradient-boosting models achieved higher overall AUPRC but low recall at the default threshold, demonstrating that performance at one classification threshold should be interpreted separately from the complete precision–recall curve.

### 4.5. Calibration Analysis

In addition to discrimination performance, probability calibration was evaluated to assess agreement between predicted mortality probabilities and observed outcomes. Calibration curves constructed using 15 equal-width predicted-probability intervals are presented in Figure 4, together with the corresponding Brier scores.

XGBoost achieved a Brier score of 0.080 (95% CI: 0.076–0.084). CatBoost also achieved a rounded Brier score of 0.080 (95% CI: 0.076–0.084), while LightGBM achieved 0.081 (95% CI: 0.077–0.084). Random Forest had a higher Brier score of 0.091 (95% CI: 0.088–0.094), whereas Logistic Regression showed the highest Brier score at 0.211 (95% CI: 0.208–0.214).

Paired bootstrap comparisons showed that the Brier score differences between XGBoost and LightGBM (p=0.296) and between XGBoost and CatBoost (p=0.741) were not statistically significant. In contrast, XGBoost had a significantly lower Brier score than Random Forest (difference = −0.0106, 95% CI: −0.0122 to −0.0090, p=0.001) and Logistic Regression (difference = −0.1304, 95% CI: −0.1351 to −0.1256, p=0.001).

Accordingly, the Brier score results support broadly comparable probability-estimation performance among XGBoost, LightGBM, and CatBoost rather than clear superiority of a single gradient-boosting classifier. These findings should be interpreted together with discrimination, precision–recall performance, the observed calibration curves, and threshold-dependent classification behavior when assessing the overall model-performance profiles.

### 4.6. Confusion Matrix Analysis

To further characterize threshold-dependent classification behavior, confusion matrices were generated for the held-out test set using the default probability threshold of 0.50 (Figure 5). The test set contained 9252 survivors and 1,068 patients with an in-hospital mortality outcome.

Logistic Regression produced 6345 true negatives, 2907 false positives, 373 false negatives, and 695 true positives. It therefore identified substantially more mortality cases than the other classifiers at the default threshold, corresponding to a recall of 0.651, but this higher sensitivity was accompanied by considerably more false-positive classifications and lower specificity (0.686).

Random Forest produced 9043 true negatives, 209 false positives, 916 false negatives, and 152 true positives. XGBoost produced 9,161 true negatives, 91 false positives, 971 false negatives, and 97 true positives. LightGBM produced 9,174 true negatives, 78 false positives, 980 false negatives, and 88 true positives, while CatBoost produced 9180 true negatives, 72 false positives, 983 false negatives, and 85 true positives.

These confusion matrices demonstrate that the gradient-boosting models strongly favored survivor classification at the default threshold of 0.50, resulting in very high specificity but low mortality-case detection. This pattern is consistent with the threshold-sensitivity analysis, which showed that lower decision thresholds substantially increased recall. XGBoost was retained as the representative model for subsequent SHAP analyses based on its numerical AUROC, AUPRC, and Brier-score performance rather than superior classification performance at the default threshold.

### 4.7. SHAP-Based Model Interpretation

After its selection as the representative model for interpretability analysis, XGBoost was examined to determine how individual predictors contributed to its mortality-risk estimates. Shapley Additive Explanations (SHAPs) were used for this purpose, assigning a contribution value to each feature for a given prediction. This approach enabled interpretation at both the cohort and individual-patient levels and provided insight into the features driving predictions from the nonlinear XGBoost model.

Global feature importance was first investigated using the SHAP summary (beeswarm) plot (Figure 6), followed by mean absolute SHAP values (Table 3 and Figure 7). Subsequently, feature interaction patterns were explored using SHAP dependence analysis (Figure 8), while patient-level prediction mechanisms were illustrated using representative SHAP waterfall plots (Figure 9). Together, these analyses provide comprehensive insight into both the overall prediction strategy of the XGBoost model and the individualized clinical factors influencing mortality prediction.

#### 4.7.1. Global Feature Importance

Figure 6 displays the SHAP beeswarm analysis for the test cohort, providing a global view of feature contributions to the XGBoost predictions. Predictors are ordered by their mean absolute SHAP values, and each point represents an individual patient. The horizontal location of a point indicates both the direction and magnitude of that feature’s contribution to the model output, while the color scale distinguishes lower feature values (blue) from higher values (red).

Mean respiratory rate emerged as the most influential feature in the XGBoost SHAP analysis, with a mean absolute SHAP value of 0.3504, followed by age (0.2670) and initial respiratory rate (0.1809). Minimum systolic blood pressure and final body temperature ranked fourth and fifth, with mean absolute SHAP values of 0.1428 and 0.1135, respectively. These rankings quantify the overall magnitude of each feature’s contribution across the evaluated cohort, whereas the direction of individual contributions is represented by the horizontal distribution of SHAP values in the beeswarm plot.

The SHAP beeswarm plot further showed that the magnitude and direction of feature contributions varied across patients. Positive SHAP values shifted the XGBoost model output toward the mortality class, whereas negative SHAP values shifted it away from the mortality class. These patterns describe the behavior of the fitted model and should not be interpreted as demonstrating causal relationships between the physiological variables and mortality.

Overall, the SHAP summary plot indicates that the XGBoost model relied primarily on cardiovascular, respiratory, and demographic features that were also prominent in the global feature-importance analysis.

#### 4.7.2. Mean Absolute SHAP Feature Importance

Mean absolute SHAP values were used to quantify the overall importance of the predictors in the XGBoost model. The corresponding feature ranking is reported in Table 3 and displayed graphically in Figure 7. Whereas the beeswarm plot shows how feature contributions vary across individual patients, the bar plot summarizes global importance by averaging the absolute SHAP magnitude of each predictor across the evaluated cohort.

Mean respiratory rate ranked first in the global SHAP importance analysis, followed by age and initial respiratory rate. Minimum systolic blood pressure ranked fourth, while final body temperature ranked fifth. Other highly ranked predictors included respiratory-rate variability, final heart rate, mean diastolic blood pressure, body-temperature summaries, mean shock index, and heart-rate summaries. These findings indicate that the XGBoost model primarily relied on respiratory, demographic, hemodynamic, and temperature-related information when estimating in-hospital mortality risk.

Additional physiological summary features also contributed to the XGBoost predictions, although their mean absolute SHAP values were smaller than those of the highest-ranked predictors. The distribution of feature importance indicates that the model integrated information from multiple first-24 h physiological summaries rather than relying exclusively on a single predictor.

The feature ordering shown in the SHAP summary plot was consistent with the ranking based on mean absolute SHAP values because both visualizations were generated from the same XGBoost SHAP results. The leading predictors consisted of demographic information and physiological summary features accumulated across the first 24 h of ICU care. This finding reflects the deliberately restricted predictor set and should not be interpreted as evidence that the model can operate during the initial phase of ICU admission or that it is equivalent to established severity-assessment frameworks.

#### 4.7.3. SHAP Dependence Analysis

To examine the relationship between a specific predictor and its contribution to mortality prediction, SHAP dependence analysis was conducted for age in the XGBoost model. In contrast to the global importance ranking, the dependence plot shows how variations in standardized age correspond to changes in the associated SHAP value.

Figure 8 presents the SHAP dependence analysis for age in the final XGBoost model. Increasing standardized age values were generally associated with progressively larger positive SHAP contributions, indicating that age increasingly shifted the model output toward the mortality class. The nonlinear pattern further suggests that the influence of age was not constant across the observed range.

Although other physiological variables were identified as important in the global SHAP analyses, Figure 8 specifically illustrates the dependence relationship for age and does not display an additional interaction variable.

Taken together, the dependence results indicate that the contribution of age to the XGBoost output varied nonlinearly across its observed range, while remaining consistent with the prominent role of age identified in the global SHAP analysis.

#### 4.7.4. Patient-Level SHAP Explanations

For patients with varying mortality risks, representative SHAP waterfall plots were created to demonstrate the interpretability of individual predictions. Unlike the global SHAP analyses described in the previous sections, waterfall plots demonstrate how each predictor contributes to the forecast for a specific patient by quantifying both positive and negative feature contributions compared to the model’s baseline prediction.

Figure 9 provides patient-level SHAP explanations for two representative predictions generated by the final XGBoost model. In the example with the higher model output, several physiological and demographic features contributed positively and shifted the prediction toward the mortality class, whereas other features exerted negative contributions. The example with the lower model output showed a different combination of feature effects, with the overall balance of SHAP contributions shifting the prediction away from the mortality class. These examples illustrate how the direction and magnitude of individual feature contributions can differ between patients even when the same prediction model is applied.

The waterfall analyses also revealed that the magnitude and direction of feature contributions differed between the representative patients. Individual predictions reflected the combined effects of several physiological and demographic variables rather than the influence of a single feature. Positive and negative SHAP contributions could therefore be examined simultaneously, providing a patient-specific view of how the XGBoost model arrived at each prediction.

The patient-level explanations were broadly aligned with the global SHAP findings presented in Figure 6, Figure 7 and Figure 8. Several predictors that ranked highly in the global analysis, including age, respiratory rate, systolic blood pressure, heart rate, body temperature, and shock index, also contributed to the representative individual predictions. This correspondence between global and local explanations shows that features with substantial cohort-level importance were also involved in shaping patient-level outputs from the XGBoost model.

Overall, the SHAP waterfall analysis demonstrates how individual feature contributions can be examined for representative patients. These patient-level explanations complement the global SHAP results and provide a more transparent view of how the XGBoost model generated individual predictions.

### 4.8. Decision Curve Analysis

**Figure 10:**
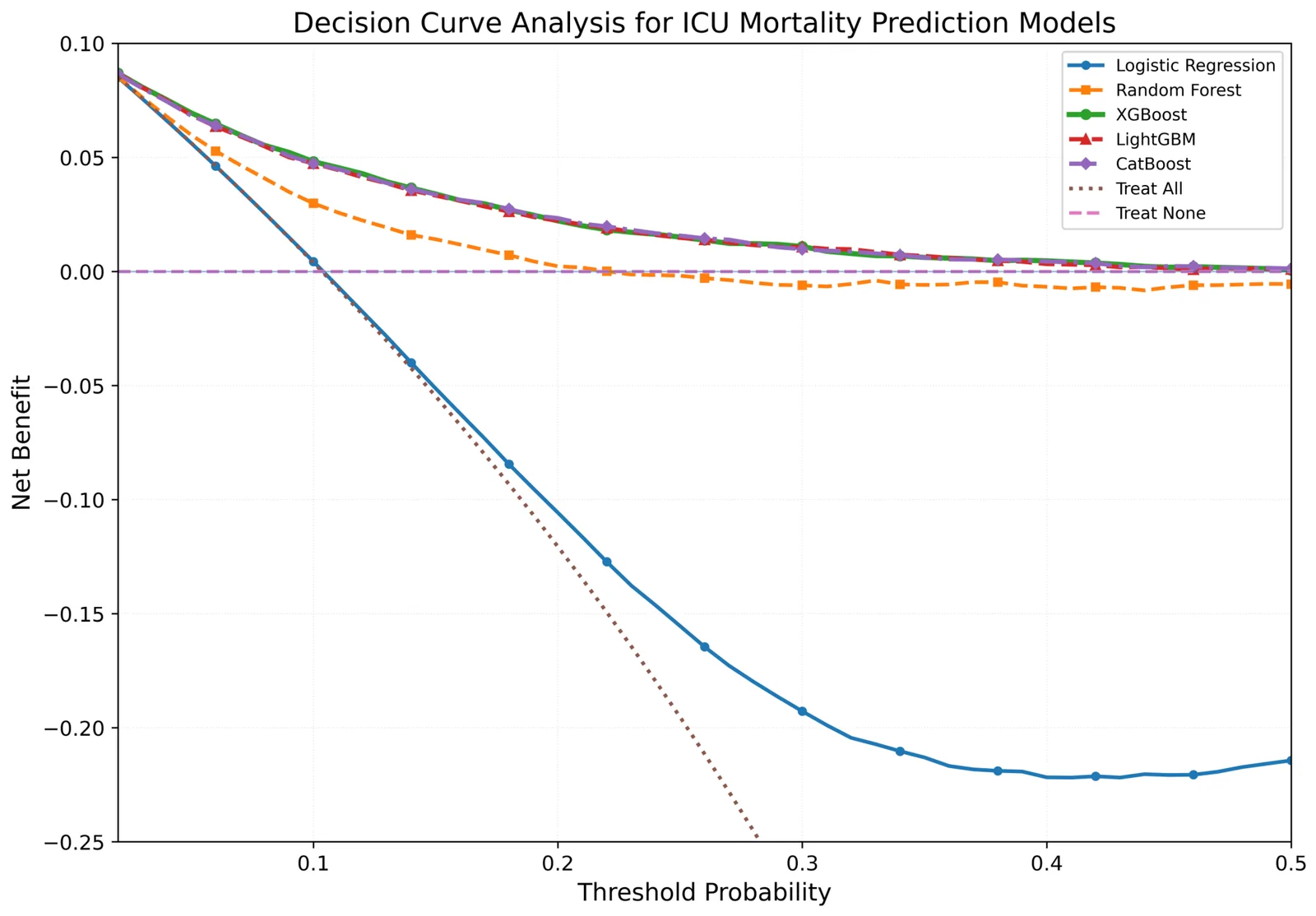
Decision curve analysis of the five machine learning models in the revised 24 h landmark cohort over the displayed threshold-probability range of 0.02 to 0.50. XGBoost, LightGBM, and CatBoost showed closely comparable positive net benefit across this range, whereas Random Forest provided lower net benefit and became negative at higher thresholds. Logistic Regression showed progressively negative net benefit as the threshold increased. The treat-all and treat-none strategies are included as reference approaches.

Decision Curve Analysis (DCA) was used to evaluate the potential net benefit of the five classifiers across a range of threshold probabilities in the revised 24 h landmark cohort. Figure 10 displays the model-specific decision curves and the treat-all and treat-none reference strategies over threshold probabilities from 0.02 to 0.50.

The three gradient-boosting models showed closely comparable decision-curve profiles. At a threshold probability of 0.10, net benefit was 0.0481 for XGBoost and 0.0474 for both LightGBM and CatBoost, compared with 0.0299 for Random Forest, 0.0043 for Logistic Regression, 0.0039 for the treat-all strategy, and 0 for treat-none. At a threshold of 0.20, XGBoost, LightGBM, and CatBoost maintained positive net benefits of 0.0222, 0.0222, and 0.0234, respectively, while Random Forest had a net benefit of 0.0023 and Logistic Regression had a negative net benefit of −0.1057.

The gradient-boosting models continued to provide positive, although progressively smaller, net benefit at higher thresholds. At a threshold of 0.30, net benefit was 0.0109 for XGBoost, 0.0108 for LightGBM, and 0.0098 for CatBoost, whereas Random Forest and Logistic Regression had negative net benefit. At a threshold of 0.50, the corresponding net benefits were 0.0006 for XGBoost, 0.0010 for LightGBM, and 0.0013 for CatBoost. These results indicate that the potential decision-analytic utility of the gradient-boosting models was greatest at lower-to-moderate threshold probabilities and diminished as the decision threshold increased.

Overall, the DCA findings showed broadly comparable net-benefit profiles among XGBoost, LightGBM, and CatBoost rather than clear superiority of a single gradient-boosting classifier. These results represent an internal decision-analytic assessment within the MIMIC-IV cohort and should not be interpreted as evidence of prospective clinical effectiveness.

## 5. Discussion

### 5.1. Principal Findings

The present study developed and internally evaluated an interpretable machine learning approach for subsequent in-hospital mortality prediction using physiological information accumulated during the first 24 h of ICU care. Among the five classifiers examined, XGBoost achieved the numerically highest AUROC (0.803) and AUPRC (0.341), together with the lowest unrounded Brier score (0.080). Paired bootstrap comparisons showed that XGBoost had a significantly higher AUROC than LightGBM but not CatBoost, while its AUPRC and Brier score did not differ significantly from either LightGBM or CatBoost. Thus, the results do not demonstrate consistent superiority of a single gradient-boosting classifier across all evaluated metrics.

Beyond predictive performance, the study incorporated SHAP-based analyses to examine how individual features contributed to the XGBoost predictions. Global importance rankings, dependence analysis, and patient-level explanations identified respiratory rate, age, systolic blood pressure, body temperature, heart rate, and shock index among the influential predictors. In the revised XGBoost SHAP analysis, mean respiratory rate showed the highest mean absolute SHAP value, followed by age and initial respiratory rate. Because these features were derived from demographic information and physiological measurements accumulated during the first 24 h of ICU admission, the resulting explanations provide an interpretable view of how first-24 h patient characteristics contributed to the model’s subsequent mortality-risk estimates.

The discovered correlations between physiological instability and mortality are congruent with recognized critical care knowledge and are clinically justifiable. Together, advanced age, tachypnea, tachycardia, hypotension, and an elevated shock index show progressive respiratory and cardiovascular decline, which are known predictors of poor outcomes in critically sick patients. These patterns are broadly consistent with established physiological indicators of deterioration in critically ill patients, although the observed associations should not be interpreted as causal.

Another important finding is that useful predictive performance was achieved using demographic information and routinely collected physiological variables. Many contemporary ICU prediction models incorporate large numbers of laboratory variables, imaging findings, medication histories, or complex deep learning architectures, which may improve predictive performance but often reduce transparency and increase implementation complexity. In contrast, the present framework uses a comparatively compact and interpretable predictor representation. However, because these predictors include summary features accumulated across the complete first 24 h observation window, the framework is intended for subsequent mortality-risk assessment after the 24 h landmark rather than rapid bedside prediction at ICU admission.

Overall, these findings suggest that interpretable machine learning models using routinely recorded physiological information can provide useful discrimination and transparent mortality-risk estimates after completion of the first 24 h ICU observation window. Such models may provide additional information to support, rather than replace, clinician judgment; however, their clinical value requires confirmation through independent external validation and prospective evaluation.

### 5.2. Comparison with Previous Studies

The present study demonstrates that interpretable gradient-boosting models based exclusively on routinely collected physiological variables can provide useful discrimination for subsequent in-hospital mortality following a 24 h ICU landmark. In the revised analysis, XGBoost achieved an AUROC of 0.803 and AUPRC of 0.341, while CatBoost and LightGBM achieved AUROCs of 0.801 and 0.798 and AUPRC values of 0.338 and 0.334, respectively. Although XGBoost had a significantly higher AUROC than LightGBM, it did not differ significantly from CatBoost for AUROC, and no significant AUPRC or Brier-score differences were observed between XGBoost and either LightGBM or CatBoost. These findings indicate small performance differences among the gradient-boosting approaches rather than consistent superiority of a single algorithm across all evaluated metrics.

Recent studies have increasingly applied machine learning to risk assessment in critical care. Aikodon et al. investigated the prediction of clinical deterioration in ICU patients using physiological information, illustrating the potential of machine learning for identifying patients at increased risk during critical care [33]. Hou et al. applied a large-scale machine learning approach to identify ICU patients with high healthcare needs and costs, providing another example of data-driven risk stratification in intensive care settings [8]. Long et al. examined ensemble-based approaches for mortality prediction in critically ill populations using retrospective cohort data and subgroup analyses [9]. Together, these studies illustrate the expanding application of machine learning to ICU risk prediction and provide relevant context for the present investigation of mortality-risk estimation following a 24 h ICU landmark.

The present study differs from more feature-intensive prediction approaches by restricting model inputs to demographic information and routinely recorded physiological measurements accumulated during the first 24 h of ICU admission. Laboratory measurements, medication histories, and multimodal clinical data were not included in the predictor set. Despite this restricted input representation, XGBoost, LightGBM, and CatBoost achieved broadly comparable predictive performance, with XGBoost showing the numerically highest AUROC and AUPRC and the lowest Brier score. XGBoost was therefore used as the representative model for the accompanying SHAP analyses, which provided both global and patient-level explanations of the features contributing to its predictions.

Model interpretability has become an increasingly important requirement for trustworthy and personalized clinical artificial intelligence [34,35]. Previous reviews have emphasized that black-box prediction models often limit clinician confidence and hinder routine clinical adoption [34]. Accordingly, the integration of SHAP within the proposed framework provides both global and patient-specific explanations that enable clinicians to understand how individual physiological variables contribute to predicted mortality risk. The observed importance of age, respiratory rate, systolic blood pressure, heart rate, body temperature, and shock index is clinically plausible and consistent with established physiological understanding of critical illness.

The intentional use of routinely monitored physiological variables rather than comprehensive severity-assessment frameworks is an important distinction of the present study. Widely used clinical scores such as the Sequential Organ Failure Assessment (SOFA) incorporate laboratory measurements and organ-function information that were not included in the present predictor set [36]. In contrast, the proposed framework uses demographic information and physiological measurements accumulated over the complete first 24 h of ICU care. Accordingly, the framework should be interpreted as a first-24 h mortality-risk assessment approach rather than a rapid or real-time prediction system operating shortly after ICU admission. Its relatively compact input structure may nevertheless facilitate future integration into electronic health record-based risk-assessment workflows after completion of the 24 h observation window.

External evaluation represents an important next step for this work. Independent critical-care resources, including the eICU Collaborative Research Database and AmsterdamUMCdb, could provide suitable cohorts for assessing whether the performance observed with MIMIC-IV is maintained across different institutions and patient populations [37,38]. Validation using such datasets would help determine the transportability and robustness of the model and should precede any prospective assessment or consideration of routine clinical use.

Finally, the present findings reinforce the growing evidence that interpretable machine learning can provide clinically meaningful decision support without sacrificing predictive performance. Rather than replacing clinician judgment, the developed model is designed to complement existing clinical assessment by providing objective and transparent estimates of mortality risk, particularly given the complexity of clinical decision-making in critically ill patients [39]. Patients identified as high risk could be prioritized for closer physiological monitoring, more frequent reassessment, earlier specialist consultation, or timely escalation of supportive care according to institutional clinical protocols.

### 5.3. Clinical Implications

The proposed framework was developed as a first-24 h mortality-risk assessment approach for adult ICU patients. It uses routinely collected physiological measurements, including heart rate, respiratory rate, blood pressure, body temperature, and a derived hemodynamic index, without requiring extensive laboratory investigations or complete clinical severity scores. Because its predictor representation includes summary features accumulated across the complete first 24 h observation window, risk estimates can be generated only after the 24 h landmark rather than during initial ICU admission.

Rather than replacing clinician judgment, the developed framework is intended to provide additional and interpretable mortality-risk information alongside existing clinical assessment. If its performance and clinical value are confirmed through independent external validation and prospective evaluation, patients assigned higher predicted risk could potentially undergo closer monitoring or more frequent reassessment according to institutional clinical protocols. However, the present retrospective internal validation does not establish that use of the model improves patient prioritization, resource allocation, treatment decisions, or clinical outcomes.

The comparatively compact input structure may facilitate future integration into electronic health record-based risk-assessment workflows because the required physiological variables are routinely recorded during ICU care. Unlike approaches that require numerous laboratory biomarkers or advanced imaging information, the proposed framework uses demographic and physiological information accumulated across the first 24 h. Accordingly, predictions can be generated after completion of the 24-h observation window, subject to independent validation and prospective evaluation before any clinical integration is considered.

SHAP-based interpretation provides an additional means of examining the model’s mortality-risk estimates. In addition to producing a predicted probability, the representative XGBoost model provides patient-level explanations showing how individual physiological features contribute positively or negatively to its output. These explanations improve the transparency of model behavior, although the present study did not evaluate whether they increase clinician confidence, improve communication, or affect clinical decision-making.

Importantly, the proposed framework should not be regarded as an autonomous diagnostic or treatment system. Clinical management decisions must continue to incorporate physician expertise, patient history, laboratory findings, imaging studies, and other relevant clinical information. Instead, the developed model is intended to provide an additional evidence-based source of information that supports, rather than replaces, clinical decision-making in intensive care practice.

Overall, the proposed framework provides a basis for further investigation as a first-24 h ICU mortality-risk assessment approach because it combines useful predictive discrimination with a relatively compact input representation and interpretable model outputs. Independent multicenter validation should first assess its transportability, followed by prospective evaluation within clinical workflows to determine whether its use affects patient management, resource utilization, or clinical outcomes before routine implementation can be considered.

### 5.4. Strengths and Limitations

Several aspects of the study strengthen its methodological design. First, model development and internal evaluation were conducted using a revised 24 h landmark cohort of 51,598 adult first ICU admissions from MIMIC-IV. The landmark design ensured that the physiological predictor summaries were constructed from a completed first-24 h observation window before the subsequent mortality-risk assessment. Second, five machine learning algorithms representing different modeling approaches were compared under a common evaluation protocol. Their performance was examined using multiple measures covering discrimination, precision–recall performance, threshold-dependent classification behavior, probability calibration, and potential clinical utility rather than being judged by a single metric. Bootstrap confidence intervals and paired statistical comparisons further quantified uncertainty in the principal performance estimates and differences between classifiers.

The emphasis on interpretability is an additional strength of this study. SHAP-based explanations were applied to the representative XGBoost model at both the global and patient levels, enabling examination of the physiological variables contributing to model output rather than treating the prediction model as a black box. Global feature importance, dependence analysis, and individualized explanations were used to characterize how the routinely collected predictors contributed to mortality-risk estimates.

Another important advantage is the exclusive use of routinely collected physiological variables accumulated during the first 24 h of ICU admission. This lightweight design reduces dependence on extensive laboratory investigations and may facilitate integration into electronic health record-based risk-assessment workflows after completion of the 24 h observation window. Because the predictor representation requires information from the complete first 24 h, the framework should not be interpreted as supporting real-time prediction at ICU admission.

Despite these strengths, this study has several limitations. First, the analysis was retrospective and relied exclusively on MIMIC-IV. Model development and evaluation used a stratified 80:20 train–test split derived from the same source database; therefore, the held-out test set represents internal validation rather than external validation. Although the test set was not used for model fitting, it originates from the same underlying healthcare system and cannot establish transportability across institutions, geographic regions, or different patterns of clinical practice. Consequently, the reported performance estimates should be interpreted as internal validation results only. Independent external evaluation in multicenter ICU cohorts, such as eICU Collaborative Research Database, AmsterdamUMCdb, or other suitable institutional datasets, is required before conclusions regarding generalizability or routine clinical application can be made.

Second, threshold-dependent classification performance represents an important limitation of the present framework. At the default probability threshold of 0.50, XGBoost achieved high specificity (0.990) but low recall (0.091), meaning that only 9.1% of mortality cases were classified as positive at this operating point. This low sensitivity would be unsuitable if the intended clinical objective were to identify most patients at risk of death. The threshold-sensitivity analysis demonstrated that lower decision thresholds substantially increased mortality detection, although at the cost of additional false-positive classifications. For example, a threshold of 0.20 increased recall to 0.434 while maintaining specificity of 0.899, whereas a threshold of 0.10 increased recall to 0.728 with specificity of 0.727. These results emphasize that the conventional 0.50 threshold should not be interpreted as a clinically optimized operating point. Selection of a clinically appropriate threshold requires consideration of the relative consequences of missed high-risk patients and false-positive alerts and should be established and prospectively evaluated in independent clinical cohorts.

Third, the present study did not perform direct comparisons with established clinical severity scores such as the Sequential Organ Failure Assessment (SOFA), Acute Physiology and Chronic Health Evaluation (APACHE), or National Early Warning Score 2 (NEWS2). The predictor set was intentionally restricted to demographic information and routinely recorded physiological measurements summarized across the first 24 h of ICU care, and it did not include the complete laboratory, organ-function, neurological, or other clinical variables required to reproduce these established scores consistently. Consequently, the present results should not be interpreted as demonstrating superiority or equivalence to SOFA, APACHE, NEWS2, or other conventional risk-assessment tools. Direct head-to-head benchmarking using cohorts in which all required score components can be reliably reconstructed represents an important objective for future work.

The compact predictor set did not include SpO_2_ or other potentially informative bedside measurements; evaluating their incremental contribution represents an important direction for future work.

Finally, the proposed framework was developed as an investigational mortality-risk assessment approach and should not be interpreted as a replacement for clinician judgment or as a clinically validated decision-support system. Mortality prediction represents only one component of comprehensive critical care management. Future work should investigate the incremental value of additional clinical information, laboratory measurements, SpO_2_, and longitudinal physiological trajectories, together with independent multicenter validation and prospective evaluation within clinical workflows.

## 6. Conclusions

Using physiological information accumulated during the first 24 h of ICU care, this study developed and internally evaluated an interpretable machine learning framework for subsequent in-hospital mortality prediction in a revised 24 h landmark cohort of 51,598 adult first ICU admissions from MIMIC-IV. Among the five evaluated classifiers, XGBoost achieved the numerically highest AUROC (0.803) and AUPRC (0.341) and the lowest unrounded Brier score (0.080). Paired bootstrap comparisons showed that XGBoost had a significantly higher AUROC than LightGBM but not CatBoost, while its AUPRC and Brier score did not differ significantly from either LightGBM or CatBoost.

At the default probability threshold of 0.50, XGBoost achieved high specificity (0.990) but low recall (0.091), demonstrating that this conventional threshold strongly favored specificity over mortality-case detection. Lower thresholds substantially increased sensitivity at the cost of additional false-positive classifications, emphasizing that the operating threshold would need to be selected according to the intended clinical use and prospectively evaluated rather than assumed from the default 0.50 value.

SHAP-based analysis of the representative XGBoost model provided population-level and patient-level explanations of the physiological features contributing to mortality-risk estimates. The compact predictor set avoids dependence on extensive laboratory profiles but should be regarded as a first-24 h risk-assessment framework rather than a real-time admission model or replacement for established clinical severity scores. Independent multicenter validation and prospective evaluation are required before routine clinical deployment can be considered.

## Figures and Tables

**Table 1:** Baseline characteristics of the revised 24-h landmark cohort. Continuous variables are presented as median [interquartile range] and categorical variables as number (percentage).

Variable	Overall	Survivors	In-Hospital Mortality	*p*-Value
Age (years)	65.00 [54.00–76.00]	65.00 [53.00–76.00]	71.00 [60.00–81.00]	<0.001
Heart rate (beats/min)	82.76 [73.41–94.04]	82.29 [73.15–93.11]	89.00 [76.69–101.89]	<0.001
Respiratory rate (breaths/min)	18.39 [16.38–21.04]	18.21 [16.27–20.68]	20.59 [17.76–24.04]	<0.001
Systolic blood pressure (mmHg)	116.00 [105.17–128.96]	116.50 [105.76–129.33]	111.00 [100.94–124.96]	<0.001
Diastolic blood pressure (mmHg)	64.54 [57.31–72.79]	64.80 [57.59–73.05]	62.22 [55.03–70.32]	<0.001
Mean arterial pressure (mmHg)	77.45 [70.11–86.33]	77.80 [70.45–86.64]	74.60 [67.63–83.19]	<0.001
Body temperature (°C)	36.82 [36.61–37.09]	36.83 [36.62–37.08]	36.79 [36.49–37.16]	<0.001
Shock index	0.71 [0.60–0.84]	0.71 [0.60–0.83]	0.79 [0.65–0.95]	<0.001
Female sex, n (%)	22,199 (43.0)	19,754 (42.7)	2445 (45.8)	<0.001
Male sex, n (%)	29,399 (57.0)	26,505 (57.3)	2894 (54.2)	–

Continuous variables were compared between survivors and patients with an in-hospital mortality outcome using the two-sided Mann–Whitney U test. Sex distribution was compared using the chi-square test. Missing observations were not included in the corresponding variable-specific descriptive summaries or statistical comparisons. Reported physiological values represent patient-level summary features calculated from measurements recorded during the first 24 h of ICU care.

**Table 2:** Comparative performance of the evaluated machine learning models for predicting in-hospital mortality in the revised 24 h landmark cohort. Classification metrics were calculated at the default probability threshold of 0.50. The best value for each metric is shown in bold.

Model	Accuracy	Balanced Acc.	Precision	Recall	Specificity	F1-Score	AUROC	AUPRC	MCC	Brier
XGBoost	0.897	0.540	0.516	0.091	0.990	0.154	**0.803**	**0.341**	0.184	**0.080**
LightGBM	0.897	0.537	0.530	0.082	**0.992**	0.143	0.798	0.334	0.179	0.081
CatBoost	**0.898**	0.536	**0.541**	0.080	**0.992**	0.139	0.801	0.338	0.179	**0.080**
Random Forest	0.891	0.560	0.421	0.142	0.977	0.213	0.795	0.323	0.198	0.091
Logistic Regression	0.682	**0.668**	0.193	**0.651**	0.686	**0.298**	0.728	0.246	**0.215**	0.211

**Table 3:** Top 20 predictors ranked according to their mean absolute SHAP values in the final XGBoost model. Higher mean absolute SHAP values indicate greater overall contribution to in-hospital mortality prediction.

Rank	Feature	Mean |SHAP|
1	Mean respiratory rate	0.3504
2	Age	0.2670
3	Initial respiratory rate	0.1809
4	Minimum systolic blood pressure	0.1428
5	Final body temperature	0.1135
6	Standard deviation of respiratory rate	0.1034
7	Final heart rate	0.0898
8	Mean diastolic blood pressure	0.0826
9	Maximum heart rate	0.0772
10	Minimum body temperature	0.0764
11	Mean body temperature	0.0751
12	Standard deviation of body temperature	0.0747
13	Final respiratory rate	0.0697
14	Standard deviation of heart rate	0.0689
15	Initial body temperature	0.0688
16	Initial heart rate	0.0686
17	Standard deviation of systolic blood pressure	0.0648
18	Mean shock index	0.0633
19	Age squared	0.0566
20	Maximum body temperature	0.0554

## Data Availability

The data supporting the findings of this study are available from the MIMIC-IV database through PhysioNet at https://physionet.org/content/mimiciv/ (accessed on 1 November 2025), subject to credentialed access and completion of the required human subjects research training. The source code used for data preprocessing, machine learning model development, evaluation, and SHAP-based analysis is available from the corresponding author upon reasonable request. A cleaned and reproducible code package is planned for public release following publication.
